# Proton Bridging in Catalysis by and Inhibition of Serine Proteases of the Blood Cascade System

**DOI:** 10.3390/life11050396

**Published:** 2021-04-27

**Authors:** Ildiko M Kovach

**Affiliations:** Department of Chemistry, The Catholic University of America, Washington, DC 20064, USA; kovach@cua.edu

**Keywords:** kinetic solvent isotope effects, proton inventories, short hydrogen bonds (SHBs), low-field, high-resolution ^1^H NMR, mechanism-based inhibitors, tight-binding or allosteric inhibitors, serine hydrolases, serine proteases

## Abstract

Inquiries into the participation of short hydrogen bonds in stabilizing transition states and intermediate states in the thrombin, factor Xa, plasmin and activated protein C–catalyzed reactions revealed that specific binding of effectors at S_n_, n = 1–4 and S’_n_, n = 1–3 and at remote exosites elicit complex patterns of hydrogen bonding and involve water networks. The methods employed that yielded these discoveries include; (1) kinetics, especially partial or full kinetic deuterium solvent isotope effects with short cognate substrates and also with the natural substrates, (2) kinetic and structural probes, particularly low-field high-resolution nuclear magnetic resonance (^1^H NMR), of mechanism-based inhibitors and substrate-mimic peptide inhibitors. Short hydrogen bonds form at the transition states of the catalytic reactions at the active site of the enzymes as they do with mechanism-based covalent inhibitors of thrombin. The emergence of short hydrogen bonds at the binding interface of effectors and thrombin at remote exosites has recently gained recognition. Herein, I describe our contribution, a confirmation of this discovery, by low-field ^1^H NMR. The principal conclusion of this review is that proton sharing at distances below the sum of van der Waals radii of the hydrogen and both donor and acceptor atoms contribute to the remarkable catalytic prowess of serine proteases of the blood clotting system and other enzymes that employ acid-base catalysis. Proton bridges also play a role in tight binding in proteins and at exosites, i.e., allosteric sites, of enzymes.

## 1. Introduction

Fundamental questions of the origins and evolutionary progress of catalytic acceleration by enzymes intrigued and motivated enzymologists for some time. This review is predicated on the vast knowledge of the serine proteases of the serine hydrolase family, especially the cardiovascular enzymes. They, like many hydrolases as cholinesterases, are of great scientific and medical interest. The catalytic acceleration of the native reactions catalyzed by these enzymes is ~13 orders of magnitude (k_cat_/k_uncat_) or greater relative to an appropriate reference reaction. The origins of this impressive catalytic power lie in sophisticated modes of transition state (TS) stabilization [1,2,3]. Proton bridges are at the heart of the catalytic prowess of serine hydrolases; when they form at the TS they lower its energy barrier, which has captured our interest. Specific structural features of the protein are decisive to the extent to which the compression of the proton bridges occurs to affect TS stabilization. The quest for understanding the role of proton bridges in catalytic efficiency required a range of biophysical chemical methods: (1) Kinetic studies included partial and full deuterium solvent isotope effects (SIEs) [1,2,4,5] and kinetic SIEs (KSIEs). (2) Structural studies included high-resolution magnetic resonance (NMR) [6,7,8,9,10,11,12,13,14] and protein dynamics (MD) calculations [15,16]. Proton bridges at the TS of serine hydrolase-catalyzed reactions have been characterized broadly by kinetic methods especially using isotope effects [1,2,3,4,5,17,18,19,20,21,22,23,24,25,26,27,28]. Our work extended to several serine proteases and cholinesterases, which revealed a dependence of the degree of participation of proton bridges in the stabilization of the TS of substrate reactions on sub-site interactions in the specificity pocket [20,21,22,23,24,25,26]. Covalent inhibitors of enzymes illuminate properties of reaction intermediates on the catalytic path. Nonetheless, another group of inhibitors bind non-covalently at the active-site region in the canonical mode and others in non-canonical modes to reveal different aspects of enzyme-substrate interactions. While catalytic sites are amenable to both kinetic and structural inquiries when using a range of rationally selected substrates and inhibitors, remote-site interactions in enzymes are particularly well revealed by structural techniques. These interactions are especially important when considering large peptide or protein substrates and inhibitors of serine proteases.

A paradigm of significant remote-site interactions presents itself in thrombin catalysis of the refashioning of its prime substrates, like fibrinogen and protein C (PC), and in its inhibition by small inhibitors that are close analogs [27,29] of the cognate substrates [30,31,32,33,34,35,36]. Factor (F) Xa-catalyzed hydrolysis of small peptides that mimic cognate substrate sites and the activation of prothrombin also reveal the participation of proton sharing in TS stabilization in the respective reactions. This is enforced at exosites and by solvate restructuring. Inhibitors offer powerful tools to study the key interactions that drive specificity and efficient catalysis, as the time window for observations is conveniently broad. Very useful has been the inhibition of thrombin by hirudin [26,37,38,39,40,41,42,43,44,45,46,47], a natural protein, and a series of its synthetic analogs [48,49,50,51,52,53,54]. The X-ray structure of the α-thrombin-sulfohirudin complex (1.84 Å resolution) revealed the occurrence of H bridges between a phenolic OH of Tyr^76^ in thrombin and an oxygen on Tys^63^ in hirudin [55]. The same oxygen atom is also in an H bond with a water molecule. An extended H-bond network connects this water molecule to another oxygen atom on the sulfate. Previous structural information from X-ray studies had revealed at least 13 sites near the cognate substrate-recognition site where hirudin might form short proton bridges. To aid locating the sites of possible short proton bridges between thrombin and hirudin, we employed several analogs of hirudin: hirunorm IV and hirunorm V [48,53,54], r-RGD-hirudin (recombinant 32SGD34 type 2 hirudin) [51] and an Nα(Me)Arg-peptide [52]. The hirunorms have three amino acids of the N-terminal sequence of hirudin followed by a linker and 10 amino acids of the C-terminus of hirudin, but are about hundred times less effective inhibitors of α-thrombin than the parent compound. One novelty in this endeavor has been the characterization of short proton bridges at binding sites near the active-site cleft and remote sites by full and partial KSIE probes and high-resolution ^1^H NMR techniques [20,21,22,23,26]. These interactions have important roles in binding of the extended substrates and inhibitors and enforcing the conformation required for efficient catalysis. The results are further supported by solvent isotope effect studies of substrate and inhibitor-binding studies [26].

## 2. Kinetic Probes of Catalysis by Serine Proteases

### 2.1. The Double Displacement Mechanism of Serine Protease Catalysis of Peptide/Protein Hydrolysis

**Scheme 1:**
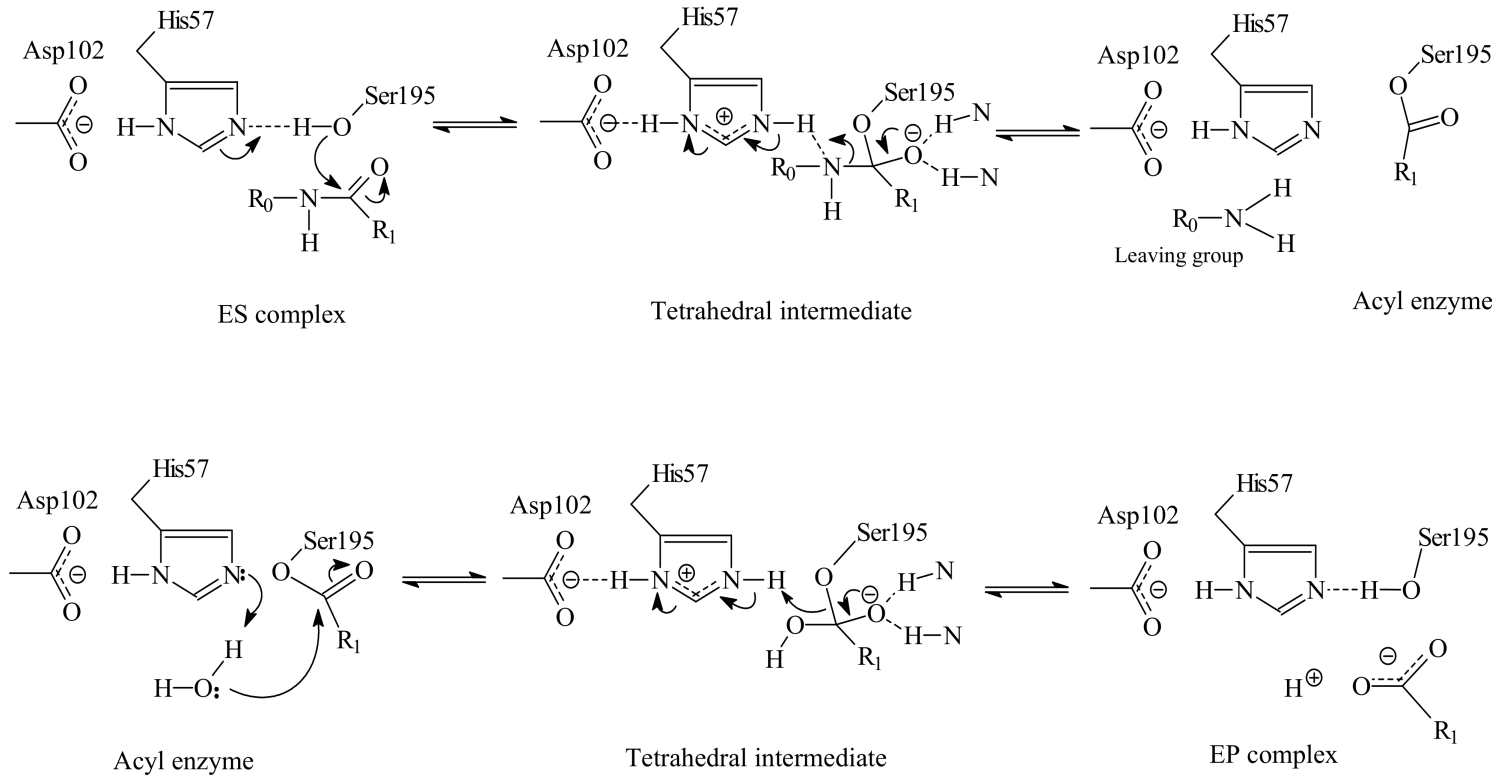
Double displacement mechanism of serine protease catalysis of peptide hydrolysis.

**Scheme 2:**
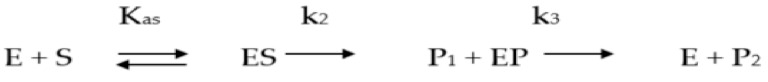
Sequence of catalytic events as binding, bond making and bond breaking.

The fundamentals of serine protease catalysis [3,17,18] are the His^57^-catalyzed nucleophilic attack of Ser^195^ resulting in the formation of a tetrahedral intermediate [56] and the ensuing His^57^H^+^-catalyzed cleavage of the C–N bond. In these general acid base-catalyzed steps, proton transfer may be mediated at short distances at the TS [2,19]. Proton sharing between the carboxylate of Asp^102^ and the imidazole of His^57^ accepting the positive charge and subsequent protonation of the leaving group can assist in lowering the TS barrier. A repeat of these steps occurs in deacylation, when water serves as nucleophile in the hydrolysis of the acyl enzyme [57]. Scheme 1 describes the molecular mechanisms of catalysis and Scheme 2 gives a general kinetic sequence of steps in the Michaelis–Menten formalism [58].

Under steady-state (Michalis Menten) conditions the constants can be defined in terms of elementary rate constants as follows: k_cat_/K_m_ = k_1_ k_2_ k_3_/(k_−1_k_2_ + k_−1_k_3_ + k_2_k_3_) and k_cat_ = k_2_ k_3_/(k_2_ + k_3_). The first step of the scheme (K_as_ = k_1_/k_−1_) is reversible substrate binding. Acylation (k_2_) follows then deacylation (k_3_). At the heart of an elucidation of the mechanisms of action of serine proteases is how the proton-transfer steps, shown in Scheme 1, are coupled or uncoupled to bond-making and -breaking at the quasi-tetrahedral TS.

### 2.2. The Catalytic Site of Serine Proteases

Proton bridges form at catalytic sites in the depth of these globular proteins to stabilize the TS. This stabilization of the TS occurs by 5–10 kcal/mol free energy provided by the acid-base catalytic machinery [1,2,3,4,5,6,14], which consist of the catalytic triad, a Ser, a His and an Asp residue [2,3,4,5,17,18,19,20,21,22,23]. Deuterium or tritium isotope effects, particularly deuterium KSIEs, proved to be great measures of the participation of protons in general acid-base catalysis by enzymes [4,17,18,19,20,21,22,23]. More in-depth information can be acquired from the proton inventory method to reveal the number and, to a degree, the nature of proton bridges at TSs [2,4,5,19,20,22,59,60,61,62,63,64,65,66,67]. R. L. Schowen and his group championed the proton inventory technique since the 1980s to unravel the role of proton bridges in a variety of serine protease. On the basis of the results of these works, and others, they promoted an understanding that interactions between S_n_ (n = 1–3) specificity sub-sites and specific amino acids in substrates at corresponding P_n_ (n = 1–3) sites elicit a compression to affect contraction of the distances between proton donors and acceptors in the acid-base machinery. A consequence is the optimization of the pK of the attacking Ser^195^ nucleophile as it covalently attaches to the carbonyl C [2,3,16]. Corroborating evidence of contraction of the critical proton bridges in the catalytic triad came from A. Frey’s group [7,68,69,70,71] and others [11,72,73,74] by measuring ^1^H NMR signals of short strong hydrogen bonds (SSHBs) in a range of serine proteases covalently modified by TS analogs. Yet, other works with substrates and inhibitors demonstrated that P’ sites [75,76,77,78] and exosites [26] in long peptide and protein substrates can have roles in exerting compression at the active site.

### 2.3. Solvent Isotope Effects and Proton Inventories 

Isotope effects originate [4,5,8,14,15,20,21,22,23,59,60,61,62,63,64,65,66,67] predominantly from a change in zero point potential energy when moving from one state to another. Deuterium isotope effects in particular stem from the mass difference of 2 between the two nuclei: They are normal when taking a value >1 because the change from reactant to TS or product results in a smaller energy gap between H and D at the TS or product state than in the reactant state; as a result, the potential energy barrier is reduced more for H than for D. In the opposite case, the isotope effect is inverse (<1). Quantum effects can become significant under certain conditions in enzymatic reactions, but only the classical treatment has been applied to these studies.

Rate ratios in water and heavy water give KSIEs of appropriate kinetic parameters. They are excellent and well-tested tools to measure the participation of acid-base catalysis in the rate-limiting process. The proton inventory is a related study of the dependence of a rate or equilibrium parameter of maximal catalytic efficiency on the composition of the mixtures of isotopic waters at the pH/pD optimum [4]. This technique aids in assessing the number of protons that participate in catalysis as transferring (readily exchangeable) protons from/to substrates and inhibitors, to/from enzymatic residues or solvating water molecules.

A rational expectation is that the most complex of catalysts, enzymes, involve more than a single protonic site in their acid-base catalytic function. The premise of the method is that the contribution of individual sites to the phenomenological solvent isotope effects can be extracted. This target can be reached by studies of rates in 5–10 different mol fractions of isotopic waters. The critical requirement for a correct and meaningful proton inventory study is a knowledge of the pH (H_2_O) and pD (D_2_O) dependence of the reaction. The rate measurements then are carried out at a pH plateau, minimum or maximum, in identically prepared H_2_O and D_2_O buffers and their mixtures [4,5,20,21,22,23,59,60,61,62,63,64,65,66,67]. The Gross–Butler equation, given below, relates the dependence of a particular rate parameter to the atom fraction of deuterium, n, in the solvent mixtures;

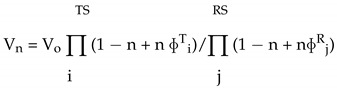
(1)
where V_n_ = velocity in a binary solvent; V_o_ = velocity in water; n = atom fraction of deuterium; RS = reactant state; ɸ^R^ = RS fractionation factor; ɸ^T^ = TS fractionation factor. (The TS sum is for TS fractionation factors and the RS sum is for RS fractionation factors.) The fractionation factors are in essence inverse equilibrium isotope effects, K_D_/K_H_, for exchange between a bulk-water site and a particular structural site of RS or TS [4,5,20,21,22,65]. The contributing isotope effects can be obtained from an appropriate least squares fitting procedure. The most common simplifications of this equation involve the assumption of a unit fractionation factor of most RSs and the assumption that one or two active-site units contribute. More complex models can also be derived from the general expression [4,5,20,21,22,57,64,65,66,67].

## 3. Structural Probes of Reaction Intermediates

### 3.1. High Resolution, Low-Field Nuclear Magnetic Resonance (^1^H NMR) Signals at Catalytic Bridges and in Binding Interactions

Spectroscopic properties of shared protons can be exploited for structural inquiries using selected inhibitors that generate covalent adducts of enzymes. H-bonding interactions in the acylation step in Scheme 1 and Scheme 2 can be modeled by mimics of the oxyanionic tetrahedral intermediate, whereas ideal models for the anionic tetrahedral intermediate in deacylation are phosphate and phosphonate ester adducts of serine proteases. The stable tetracovalent adducts of enzymes and TS analogue adducts with inhibitors can be studied by high-resolution ^1^H NMR at low field. Short proton bridges manifest in the ^1^H NMR spectra as a unique resonance between 14 and 21 ppm downfield from silanes [7,8,9,10,11,12,13,14]. The low-field signals can also be observed below pH 6 with some native enzymes that operate acid-base catalysis [7,8,9,10,11,12,13,14,23,24,25,26]. The deshielding phenomenon has been attributed to the presence of short strong H-bonds (SSHBs) at the active site of enzymes as the key base catalyst (His) becomes protonated. The deshielding is due to the loss of some of the sigma bond electron density upon lengthening slightly the H-donor distance. SSHBs have been detected [14,68,69,70,71,72,73,74] by ^1^H NMR methods in several enzymes, other than serine proteases. These cases satisfy four criteria; (a) the occurrence of highly deshielded proton resonances (below 14 ppm), (b) lower than one D/H fractionation factor, (c) at least 10 times slower exchange rates than in average H-bonds, (d) SSHBs ~5–10 kcal/mol stronger than normal proton bridges as measured by pH titration or by the deductions from the values of kinetic parameters in active-site mutants [7,8,11,79,80,81,82,83]. However, earlier estimates of the unusual bond strength have not been substantiated and to recognize this fact the notation of short H-bond (SHB) will be used in the following [82,83,84,85]. From ^1^H NMR data, the SHB length of 2.4–2.7 Å and donor-H-acceptor angles can be calculated. In contrast, normal H-bonds in proteins and nucleic acids are 2.7–3.0 Å in length and ~1–2 kcal/mol in strength [85]. These small differences in H-bond distances are frequently not discernible in X-ray structures. Protons can be located reliably by neutron diffraction spectroscopy which is still rarely available [14,79,80,81,85]. Mildvan [14] offered an alternative using two methods for the calculation of proton donor-acceptor distances; from chemical shifts and D/H fractionation factors of the shared protons.

### 3.2. Computational Studies: Molecular Dynamics

Random thermal fluctuations of enzymes that keep them in constant motion result in many conformers that differ slightly in energy [86]. These different conformations offer different epitopes for binding, i.e., different surface interactions with cognate substrates and other effectors [86,87,88,89]. It appears that some of these motions result in compression of critical distances to enforce a catalytic reaction. α-Thrombin as an allosteric enzyme fulfills its various functions in response to stimuli by adopting different conformations for the binding of its effectors [47,90,91,92]. Molecular dynamics (MD) simulations of these motions in the presence and absence of effectors are very revealing techniques.

For example, we performed MD simulations with CHARMM [93] of the interactions between components of the enzyme active site and a modifier to shed light on experimentally inaccessible questions involving transients [94,95]. Differences have been observed between acetylcholinesterase and trypsin or chymotrypsin in the use of the catalytic triad, oxy anion hole and other active-site components in tetrahedral carbonyl and tetra and pentacoordinate phosphonate adducts of the enzymes [94,95,96].

## 4. Basic Characteristics of the Enzymes of Bold Clotting Supporting the Catalytic Function

α-Thrombin and FXa are key serine proteases in hemostasis and thrombin also plays a role in thrombolysis [30,31,32,33,34]. Other serine proteases in blood coagulation are factors VIIa, IXa, Xa, XIa, XIIa and PC. Plasmin, and plasminogen activator enzyme are serine protease enzymes with thrombolytic function [30,31,32,33,34]. Plasmin efficiently degrades fibrin that forms the meshwork of a thrombus and thus provides an important counterbalance to the blood coagulation cascade in vivo [30,31,33]. The coagulation enzymes are two/four-chain glycoproteins with modular structures [30,33] in which components have high specificity for binding different macromolecules [35,36,37,38,39,47,97,98,99]. The enzymes become activated from their zymogens by a serine protease-catalyzed, Ca^2+^ ion, cofactor and plasma-membrane-dependent cleavage of one or more Arg-Gly (Val, Ilu, Ala, Leu or Asp) bonds [35]. Individuals who lack the presence or the vitamin K-dependent activation of the zymogens, factors V, VII, VIII, IX, or X, suffer from profound hemostatic defects and those with deficient FXI also show some of the defects [33]. Activated PC (APC) is an anticoagulant, thus, PC deficiency is a thrombotic risk [35].

The enzymes execute their roles very selectively only with physiologic substrates and predominantly in the presence of regulatory molecules. Binding of substrates and inhibitors at P sites, P’sites and exosites may have complementary or compensatory roles [75,76,77,99]. The most extensive mechanistic information is now available for thrombin [32,35,36,37,38,51,55,88,89,100,101,102,103], but the understanding of the mode of operation of both FXa [33,104,105] and plasmin [33] are also increasing rapidly. The availability of crystal structures of thrombin [32,88,89,100,101,102] and FXa [105] now provide the basis for interpretation of kinetic data at the molecular level or even in atomic detail.

Processing of thrombin occurs when FXa catalyzes the hydrolysis of two peptide bonds in the zymogen. In vivo, the prothrombin complex includes prothrombin, Ca^2+^, and FVa bound to a phospholipid surface where two bonds, Arg^322^-Ile^323^ and Arg^273^-Thr^274^, 36 Å apart, are cleaved by FXa in succession. If FVa is left out of the complex, the cleavage rate drops by five orders of magnitude and it occurs in the opposite order [105]. If the construct forms correctly, thrombin production is channeled without the release of intermediates. In this process an exosite of FXa becomes exposed near the active site, which seems to serve as a docking site of the scissile bond on prothrombin. This exosite is critical for the correct orientation of the Arg^322^ cleavage site. Cleavage is preceded by a rotation around two Gly residues hinge points. The presence of FVa promotes the first bond fission but the second one seems unaffected. Interestingly, FVa itself is processed from its precursor by thrombin-catalyzed cleavage at Arg^709^ into a heavy and a light chain.

One distinctive difference in the active-site composition of the enzymes is the presence of Glu^192^ in thrombin and PC, which is Gln^192^ in FXa and FVIIa [106]. This difference in the negative electrostatic medium at the active site is the origin of discrimination between natural inhibitors and chromogenic substrates; Glu^192^ confers resistance to Kunitz type inhibitors [102] whereas FXa is prone to inhibition by them. Such phenomena were studied with the Q192E mutant of FXa [106,107,108] and the E192Q mutant of thrombin [88,105]. The E192Q mutation in thrombin resulted in higher cleavage rates of substrates that have an acidic residue at P_3_. In the regulatory role of thrombin, thrombomodulin (TM) induces a conformational change that may move Glu^192^ in the catalytic pocket of thrombin out of the way of PC binding. Our interest was piqued by the great importance of the absence of negative charge at 192 in FXa in the process of prothrombin activation as most of the P_3_ and P_3_’ sites in human and bovine prothrombin are acidic. An important study of the activation of prothrombin by FXa and its Q192E mutant in the presence and absence of FVa indicated that FVa can compensate for the electrostatic repulsion between the Q192E mutant and the acidic P_3_ and P_3_’ sites [106].

α-Thrombin is probably the most selective and multifunctional of the serine proteases recruited in blood clotting; it plays a role in both hemostasis and thrombolysis [88,89,109,110,111,112,113,114]. It cleaves 12 substrates with specific cofactors. Activation of fibrinogen to fibrin is the most broadly studied among the natural reactions, which assisted our investigations into the role of SHBs in this vital reaction. It occurs in distinct steps in a sequential mechanism producing FpA and FpB to be further processed to fibrin. Thrombin is down regulated by its own action when it activates PC. Thrombin is a remarkable catalyst owing to the unique combination of catalytic features: the catalytic triad, the oxyanion hole, and two specificity-binding, I and II, exosites. While the latter may vary significantly among the members of this large class of enzymes, they all perform the task of nucleophilic displacement at carbonyl by the double displacement mechanism, shown in Scheme 1 [2,3,17,18].

### 4.1. The Catalytic Mechanism of α-Thrombin and the Role of Water Networks

The catalytic machinery (Ser^195^ His^57^ and Asp^102^), is at the bottom of the active-site cleft of α-thrombin [87,88,89,100,101,102]. The pK of His^57^ in free thrombin was reported to be 6.7 ± 0.3 and two other ionizable residues with pK 8.4 ± 0.4 and ~8.0 were identified at µ = 0.1 and 25 °C [115,116,117,118]. The second pK was assigned to the breakage of the salt bridge involving Ilu^16^ and the third one was attributed to a group located in the fibrinogen recognition (exo) site (FRS). The first two pKs change upon binding of small peptide substrates to 6.16 ± 0.25 and 8.95 ± 0.41, respectively, while the third pK remains the same. Higher pKs but similar trends were also reported for the thrombin-catalyzed fibrinogen activation and oligopeptide hydrolysis reflecting a complex dependence on ionizing groups under different conditions [35,114]. Similar kinetic pKs, 6.9 and 8.8, were reported for the reaction of FXa with specific ester substrates [119].

A prescient suggestion of Lottenberg et al. [118] that the unique pH dependence of the thrombin-catalyzed hydrolysis of a series of oligopeptide substrates can be explained by two or three protons participating in the mechanism guided our inquiry. Sizable solvent isotope effects were reported by Stone et al. [120] supported the anticipation of multi-proton catalysis by thrombin, FXa, APC and plasmin, if the requirements for optimal interactions between enzyme and substrate subsites were satisfied. In fact, these systems provide the most relevant cases for exosite-dependent and independent P’ specificity and their effect on proton participation in TS stabilization.

Catalysis by α-thrombin is controlled at several proton-linked ionizable groups [87,88,89,109,110,111,112,113,114,115,116]. The studies that informed our investigations were on the elucidation of the mechanism of allosteric regulation of α-thrombin using spectroscopic and kinetic measurements. These studies led to the proposition of the “fast-slow” conformational transition [87,88]; however, a general induced fit model can also account for the conformational flexibility of thrombin [89]. Di Cera’s proposition is that α-thrombin exists in either of the fast or the slow form, but only the fast form binds Na^+^ ion whereby it activates α-thrombin. Many peptide substrates and fibrinogen recognize and bind the fast conformation in the procoagulation process, whereas preferential binding to the slow conformation, or induction of the fast-slow conformational change, is associated with the anticoagulant role of thrombin [112,113,114]. The allosteric effector binds to the enzyme and affects its kinetic properties; a linkage effect. For example, fibrinogen binds to the FRS first then Na^+^ ion bound in its pore facilitates binding of the rest of fibrinogen by forming the link through the water channels to the active site. As these systems often disobey Michaelis–Menten kinetics [88,109,110], a comprehensive mathematical treatment of linkage thermodynamics and kinetics was applied to the regulation of α-thrombin by macromolecules and by the presence or absence of Na^+^ ions [113] and other monovalent cations [112,113]. Na^+^ ions, abundant in the extracellular medium, are the most effective in stabilizing the conformation of α-thrombin that accommodates peptide substrates and fibrinogen [87,88]. Na^+^ ion binding occurs on a faster time scale than the slow-fast transition [87,88,121,122]. This was elucidated from fast kinetic measurements of Na^+^ ion binding using the augmentation of the intrinsic fluorescence of thrombin [121]. The Trp residues of α-thrombin giving rise to its fluorescence were eliminated successively by site-specific mutations to map the exact role of each Trp in the intrinsic fluorescence of α-thrombin [121]. These studies revealed that a key contributor to fluorescence is Trp^215^ at the aryl binding site. The fluorescence studies showed that the Na^+^ ions bind to α-thrombin at about three orders of magnitude slower than many natural substrates do [121,122]. The reason for this is the need for stripping off water hydrate from Na^+^ ions to enable their penetration into the pore defined by loops 186- and 220- on α-thrombin [87,123]. After entering its site, the Na^+^ ion is coordinated with the backbone oxygen atoms of Arg^221^, Lys^224^ and four water molecules [87,88]. Ensuing this event is a grand organization of the water network that spans the interior of α-thrombin in a length of 15 Å leading up to the catalytic Ser^195^. This entire process of Na^+^ ion binding including the organization of water channels apparently slows down the association of Na^+^ with α-thrombin. An important physiological ramification of Na^+^ ion binding is that natural mutations in the prothrombin gene affecting residues of the Na^+^ ion binding site often result in bleeding [87,121].

Huntington [47,89,124,125] proposed a flexible, the plastic, model of thrombin on the basis of 2 dimensional ^1^H ^15^N NMR and molecular dynamics investigations. His group analyzed vastly different conformations of unliganded thrombin structures or zymogen like states, which yielded solution-state structures. Their fundamental finding is that thrombin is inherently flexible at the sites of binding and activity as it rapidly samples multiple states corresponding to the slow form or zymogen like states. Thrombin’s recognition of a number of binding sites of its multiple substrates and cofactors are key to its unique catalytic role at the end of the blood cascade system. Ligands bind through induced fit or cofactor-triggered conformation at active sites and exosites.

### 4.2. Thrombin Inhibition by the Hirudin Family

Substrates like fibrinogen or PC exploit numerous remote-site interactions with thrombin that can be sampled indirectly with large substrate-mimic inhibitors, which use the same epitopes as one or the other substrate. Hirudin and its analogs are tight-binding inhibitors that utilize extensive interactions at subsites and two exosites, primarily at the FRS, but without forming covalent bonds [86]. H bonds, some with the potential for an SHB, are numerous among these interactions [86].

Hirudin is a 65-residue protein produced in the salivary glands of the common leech, *Hirudo medicinalis*, in three varieties of close sequence homology [39,46,126]. All three variants contain three disulfide bonds and a sulfated Tyr^63^, Tys^63^. *Hirudo aiponnia* and *Hirudinaria manillenis* produce other isoforms of hirudin that contain an Asp residue instead of Tys^63^ [126]. Hirudin interacts non-covalently but tightly with α-thrombin within the active-site cleft as well as with the FRS [46,127,128,129]. It is an allosteric effector of the fast conformation of α-thrombin. The first X-ray structure (2.3 Å) of the α-thrombin-r-hirudin complex (variant 2, Lys^47^) afforded a complex picture of the key interactions [128,129,130,131]. Three residues of the N-terminal, Ile^1′^-Val^2′^-Tyr^3′^, penetrate the active site and aryl binding site where they interact with the S_1_ specificity site and form H bonds to His^57^ as well as Ser^214^ in thrombin. The central portion is globular and more loosely attached to α-thrombin. The N-terminal head of r-hirudin forms a parallel β-strand with thrombin (214–219) making a non-substrate like interaction. The 53–65 C-terminal fragment of hirudin binds the tightest to residues 62–73 of the B-chain on α-thrombin. Strong electrostatic interactions including at least 13 H bonds hold this segment together, but the last five residues form a 3_10_ helical turn, which engages in hydrophobic interactions. Native hirudin with the sulfate group on Tys^63^, enhances the binding constant by ~20-fold over the desulfo form [46,55].

The intrinsic fluorescence of α-thrombin has been employed for measurements of binding parameters, because r-hirudin binding causes key Trp residues bury more deeply in the interior and thus enhance fluorescence [132]. Tt emerged from two studies [112,132], that first the C-terminal segment is preoriented and binds rapidly to the FRS because of the complementary electrostatic forces between the two. This is followed by the fitting of the N-terminal segment, which is ~300 times slower than the first step. The N-terminal fragment (1–52) and a C-terminal fragment were also used in this study to elucidate the binding events and calculate rate constants [132].

Several analogs were fashioned on the full hirudin chain including hirulogs [133] and hirutonin to include an active-site-directed N-terminal, a spacer of some length and the C-terminal hirudin tail or a variant of it. Hirunorms [48,49,53,54] were designed to be effective hirudin mimics by containing the functionalities that interact with the α-thrombin active site, specifically the Ser^214^-Gly^216^ segment, and with the FRS like hirudin does [46]. A three-residue segment consisting of D-Ala^6″^-βAla^7″^-βAla^8″^ or D-Ala^6″^-Gly^7″^-βAla^8″^ was used as a spacer in place of the larger Cys^6′^-Lys^47′^ core in hirudin. Hirunorms IV and V were reported to be the most potent among five hirunorms. X-ray structures of α-thrombin-hirunorm IV [54] and α-thrombin-hirunorm V complexes [53] show that the hirunorms interact along the B-chain blocking the active-site cleft by interacting with key residues in a parallel manner and stretch out of the cleft and around, so that the C-terminal interacts with the FRS. The primary sequence of hirunorms IV and V differ only at the second residue and only slightly along the C-terminal. The H-bonding potential between α-thrombin and the C-terminal of these inhibitors is similar to that of hirudin, but they bind with ~3 kcal/mol less energy than hirudin.

Our endeavors built on the above discoveries as we embarked on interrogating the effect of binding interactions at binding sites and exosites, on the formation and strength of SHBs at active sites in TS stabilization and at binding sites in cardiovascular enzymes. As shown above, these enzymes are unique in their great specificity and allosteric use of exosites, beyond S and S’ binding sites. The investigations included reaction dynamics and structural stabilization of intermediates. A great medical significance of the enzymes was additional justification of our quest [30,31,32,33]. Experimental details can be found in an Appendix A and in references [20,21,22,23,26,134,135,136,137,138].

## 5. The Occurrence of Short Hydrogen Bonds (SHBs) in Catalysis by and Inhibition of Blood-Clotting Enzymes

### 5.1. Probing the Dependence of Proton Sharing at the Transition States on Subsite Binding of Substrates

Lottenberg et al. [118] suggested that the unique pH dependence of the thrombin-catalyzed hydrolysis of a series of oligopeptide substrates can be explained by two or three protons participating in the mechanism. KSIEs > 3 were reported by Stone et al. [120] which lent support to the anticipation of multi-proton catalysis by thrombin, FXa, APC and plasmin, if the requirements for optimal interactions between enzyme and substrate subsites were satisfied. A proton inventory study had been completed for the thrombin-catalyzed hydrolysis of the minimum substrate Z-Arg-ethyl ester [4,65]. Not unexpectedly, this reaction involves one-proton catalysis. Overall, the conclusions from our studies fully concur with these earlier results and predictions. Moreover, the results obtained with natural substrates, indicate that solvent rearrangement in association and dissociation steps is manifest in inverse or very inverse KSIEs.

We constructed full and partial solvent isotope effect profiles reporting on a great number of protonic sites and their mode of participation in the catalysis of substrate hydrolysis by the four enzymes [20,21,22]. The substrates were selected to model cognate specificity subsites. Substrates were three types: (1) Chromogenic or fluorogenic di- to tetrapetide amide substrates test the effect of P_1_-P_4_ residues. (2) To test the collective contribution of P_1_-P_4_ and P_1_’-P_3_’sites without exosites, fluorescence-quenched substrates with an N-terminal 2-aminobenzoyl (AB)-Val fluorophore, a C-terminal Lys-2,4-dinitrophenyl (DNP) quencher, and an Asp-OH to enhance solubility, were studied with thrombin. (3) The effect on the extent of protonic participation of exosites (other than the N-acyl, or leaving group-binding site) were evaluated from studies of selected natural reactions. Naturally occurring substrates of α-thrombin and FXa achieve specific binding to the enzymes at designated exosites remote from the active site. Remote site interactions are expected to modify other, particularly P’-site, interactions. Enzyme–substrate pairs were studied as follows

*Thrombin:* (1) Z-Pro-Arg-7-amido-4-methylcoumarin (7-AMC) (PR-AMC); N-t-Boc-Val-Pro-Arg-7-AMC (VPR-AMC); Bz-Phe-Val-Arg-p-nitroanilide (pNA) (FVR-pNA); and H-D-Phe-L-Pip-Arg-pNA (FPiR-pNA). (2) Internally quenched fluorogenic peptides; (a) the optimal substrate; (AB)Val-Phe-Pro-Arg-Ser-Phe- Arg-Leu- Lys(DNP)-Asp-OH, (P_1_-P_3_-P_1′_-P_3′_ = FPR-SFR) and (b) a recognition sequence for FVIII; (AB)Val-Ser-Pro-Arg-Ser-Phe-Gln-Lys(DNP)-Asp-OH, (P_1_-P_3_-P_1′_-P_3′_ = SPR-SFQ) [10,88,89]. The hydrolysis of N-t-Val-Pro-Arg-7-AMC catalyzed by thrombin demonstrated that fluorescence quenching by the substrate limited the useful range of substrate concentration as the mmol range was approached. Quenching by a substrate, of the fluorescence of a reaction product was quantitated under a set of conditions and a correction was applied to the slope for the initial rate measurements. (3) Natural substrates; (a) fibrinogen to fibrin, FpA and FpB by high-performance liquid chromatography (HPLC) and blood clotting curves developing after the release of FpA; (b) the activation of PC to APC.

FXa: (1) N-α-Z-D-Arg-Gly-Arg-pNA (RGR-pNA); H-D-Ile-L-Pro-L-Arg-pNA (IPR-pNA. (2) Prothrombin activation to thrombin.

*Plasmin:* Pyr-Glu-Phe-Lys-pNA (EFK-pNA); H-D-Val-Phe-Lys-pNA, (VFK-pNA); H-D-Val-Leu-Lys-pNA (VLK-pNA).

*APC:* H-D-Ile-Pro-Arg-pNA (IPR-pNA); Pyr-Glu-Pro-Arg-pNA (EPR-pNA).

Michaelis–Menten parameters of the reactions were characterized as a function of pH and dependence on NaCl concentrations at 25.0 ± 0.1 °C. The optimal pH was above 8.4 for chromogenic and fluorogenic substrates of thrombin and at 8.0 for all other reactions.

It has been shown [3,17,18,19,57] that the deacylation manifold is faster than acylation in serine protease-catalyzed hydrolysis of amides and peptides and, thus, the rate-determining step is generally in the acylation manifold. Strongly bound or poorly aligned leaving groups can present barriers. Accordingly, it is proton-transfer assisted either C–O bond formation to Ser^195^ and/or bond fission to the leaving group that is typically revealed by these studies. In terms of elementary rate constants, since k_2_ < k_3_, k_cat_/K_m_ = k_1_k_2_/(k_−1_ + k_2_) and k_cat_ = k_2._, The rate of reaction occurs near diffusion control at low substrate concentrations, i.e., k_cat_/K_m_ = k_1_ [139], in the reactions of highly developed enzymes with very efficient, as the natural, substrates. Because the isotopically sensitive step is concealed, only a small isotope effect for encounter (~1.17) is expected. However, if conformational adjustments accompany encounter many small inverse isotope effects result. These can add up to values 0.3–0.9 [4,5,20,22].

Initial rates were measured or full progress curves constructed for the above systems at the optimal pH and at 25.0 ± 0.1 °C. Some k_cat_/K_m_ values approach 10^8^ M^−1^ s^−1^ as was reported for k_1_ the rate constant for association of the best substrates with thrombin. P-nitroanilide tripeptides give the highest reaction rate constants. There are four orders of magnitude difference in the value of the kinetic parameters from the simple PR-AMC substrate to the most efficient FPiR-pNA substrates of thrombin. The kinetic parameters obtained [20,22] reflect on the compliance of the subsites, especially in the S_3_ location. The lack of a P_3_ residue in PR-AMC results in a large K_m_ and a small k_cat_ values. Somewhat unexpected is that the decapeptide substrate of thrombin does not bind better than PR-AMC does, which is likely a consequence of the compromised fit of the leaving group segment. The disadvantage is compensated for by a great k_cat_ value and, thus, a large bimolecular rate constant value. The encounter of thrombin and the decapeptide occurs at near the rate of diffusion.

An extensive statistical analysis of the proton inventories involved fitting of 6–8 mathematical models to the data: adaptations of the Gross–Butler equation of the five most useful models are shown in Chart 1 [4,5,20,22].

In the Chart 1, k_n_ represents an observed rate constant, V_max_ or V_max_/K_m_ (k_cat_ or k_cat_/K_m_) determined at n > 0 and k_H_ represents rate constants determined in water (n = 0). The fractionation factor for the reactant state was set to RS = 1. (TS fractionation factors, ɸ*_n_*, and an exponential term for H-bond contributions from water (s^n^) were the fitting parameters.) The calculations were performed with each proton inventory study and the model with the smallest value of χ^2^ yielding self-consistent fractionation factors between V_max_ and V_max_/K_m_ measurements were identified. Specific models are shown for protonic participation at the TS_i_s and solvent (solv) of the rate-determining step. A general solvation term was calculated as an exponential dependence on n.

As the D/H fractionation factor (ɸ) calculated for an H-bond is an equilibrium constant for the exchange of H for D between a site on a protein and the protic solvent, i.e., L_2_O (L = H, D), it can be written as; ɸ = [Active site-D][Hsolvent]/[Active site-H][Dsolvent]. This expresses the preference of the active site for D over H in reference to the solvent, i.e., an inverse deuterium solvent isotope effect [4,5,19,20,59,65]. The shorter and stronger the H-bond, the smaller the value of ɸ. The classical representation of the potential energy of the proton vibration in the proton bridge is a double well model, in which the minima in the wells are centered at one covalent bond length from the donor and acceptor heavy atoms. As the donor and acceptor atoms move closer to each other, the wells approach each other and become broader and shallower. The origin of the difference between proton and deuteron transfer at the TS lies in the loss of the difference in zero point energies existing in the ground state vibration of H/D in bonds to other atoms. Due to the 2-fold greater mass of a deuteron than a proton, its zero point vibrational energy decreases (2)^1/2^ –times, which results in a decreased preference for deuterium over protium (ɸ). An estimate may be made from the value of ɸ of the length of the SHB by empirical methods. However, a refined approach may involve quantum effects or polarization effects. Electronic and nuclear quantum effects are generally small and undetectable by classical methods, but unique to SHBs unlike in H-bonds with longer bonds.

In most of the reactions studied, it is assumed that the rate-limiting step is acylation for both the k_cat_/K_m_ and the k_cat_ terms and, thus, the fractionation factor for proton transfer at the TS should be the same for the two terms but the contribution of solvation is usually quite different. Ostensibly, the solvation term is important in binding and not at all in the catalytic chemical step. Due to the complex contribution of solvation, which can have either a normal or an inverse overall SIE, the shape of the proton inventory for the k_cat_/K_m_ term is frequently bowl shaped or dome shaped.

**Figure 1:**
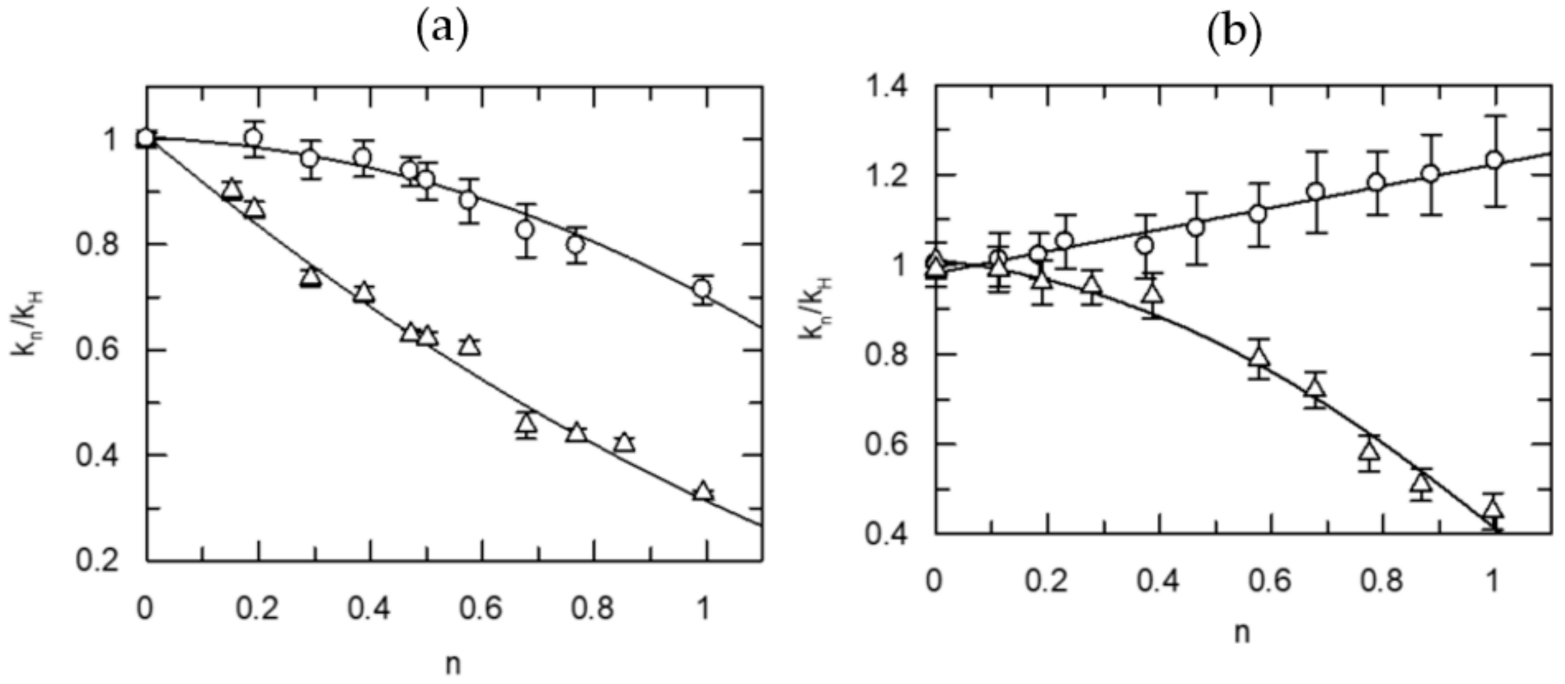
Proton inventory for the thrombin-catalyzed hydrolysis of, (**a**) left side, VPR7-AMC (N-t-Boc-Val-Pro-Arg-7-AMC) and, (**b**) right side, FPR-SFR in 0.02 M Tris buffer, pH 8.4 containing 0.3 M NaCl, 1% DMSO, 0.1% PEG-4000 at 26.0 ± 0.1 °C. Circles represent k_cat_/K_m_ and triangles k_cat_. Curves were calculated from the best-fit models in Table 1, as introduced in Chart 1.

Table 1 illustrates selected numerical data for the most plausible models, which gave the best statistical results as measured by the χ^2^ and F tests for the proton inventory study of the thrombin-catalyzed hydrolysis of N-t-Val-Pro-Arg-7-AMC (VPR-7-AMC). The data for the model of choice based on the goodness of fit, consistence and a sensible mechanism, are printed in bold face letters in Table 1. Figure 1 shows the proton inventory curve on the left side. On the right side of Figure 1 is a representative curve for an internally fluorescence-quenched substrate, (AB)Val-Phe-Pro-Arg-Ser-Phe-Arg-Leu-Lys(DNP)-Asp-OH, (P_1_-P_3_-P_1′_-P_3′_ = FPR-SFR).

It is noteworthy that almost identical TS fractionation factors were calculated for the two-proton transfer plus solvation model, under k_cat_ as well as k_cat_/K_m_ conditions, consistent with the proposition that acylation is the rate-determining chemical step for both Michaelis–Menten parameters of this reaction. For k_cat_, fractionation factors for TS_1_ and TS_2_ were 0.57 (isotope effect = 1.9 for each proton transfer) while they were constrained to be equal whether solvation was calculated or not. That is, the fractionation factor for solvation was calculated to be 1.0 within experimental error. There was a slight difference between the two fractionation factors when the contribution of solvent was constrained to 1.0 but ɸ_1_ and ɸ_2_ were unconstrained. Nearly the same value, 0.55, was obtained for the k_cat_/K_m_ term when the values of the fractionation factors for TS_1_ and TS_2_ were constrained to be equal, but the contribution of solvent was calculated. As the up-bulging curve for the dependence of the k_cat_/K_m_ isotope effects on n suggests, there is a strong contribution of solvent reorganization at the TS for this term. The deduced data are most consistent with two protons participating coupled to either the formation or breakdown of the tetrahedral adduct in the rate-determining acylation. In the spirit of the mechanism depicted in Scheme 1, the catalytic protons are between Ser^195^-His^57^ and His^57^ and Asp^102^. Solvent reorganization occurring at the TS for acylation masks the contribution of the proton-transfer steps under k_cat_/K_m_ conditions in the overall solvent isotope effect, but the proton inventory analysis aids in unravelling the contribution and nature of elementary steps.

**Figure 2:**
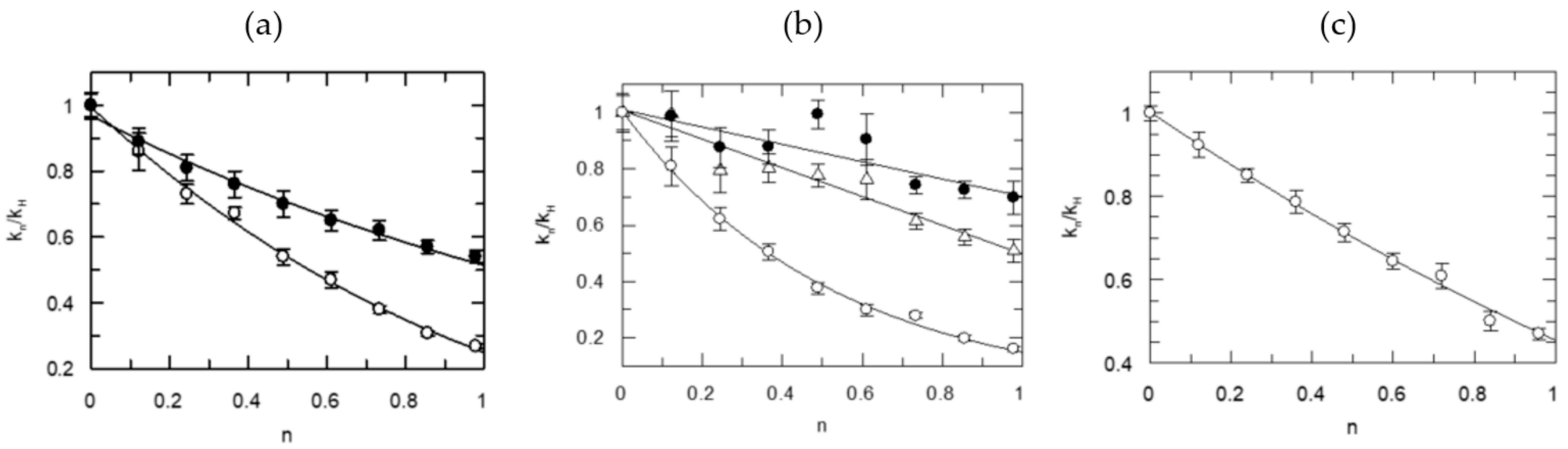
Proton inventories for the 1.45 × 10^–8^ M bovine FXa-catalyzed hydrolysis reactions, at pH 8.4 and equivalent pL (L = H, D and their mixtures), 0.02 M Tris buffer, 0.30 M NaCl, 5 × 10^−3^ M CaCl_2,_ 0.1% PEG4000, at 25.0 ± 0.1 °C: (**a**) Left side: 1 × 10^–3^ M RGR-pNA, k_cat_ open circles, the equation of the line is k_n_/k_H_ = 1.0 (1 − n + n/1.75)^2^ 0.78^n^; k_cat_/K_m_ closed circles, the equation of the line is k_n_/k_H_ = 1.0 (1 − n + n/1.37)^2^. (**b**) Center: 1 × 10^–3^ M RGR-pNA elementary rate constants were calculated from progress curves as K_1_ = k_1_/k_−1,_ triangles, k_2_ closed circles, and k_3_ open circles. The equations of the lines for fitting to the data are: k_n_/k_H_ = (1 − n + n/(2.03)); k_n_/k_H_ = (1 − n + n/(1.43)); and k_n_/k_H_ = (1 − n + n/(3.11)^2^), respectively. (**c**) Right side: IPR-pNA; ratios of initial rates measured under saturating levels of substrate and plotted against *n* and the equation of the line is k_n_/k_H_ = 1.0 (1 − n + n/1.5)^2^ (See Chart 1).

Figure 2 offers another example, the proton inventory results for two FXa-catalyzed reactions. The data for the Michaelis–Menten parameters are plotted on the left side. On the center graph, the resolution of the phenomenological rate constants into elementary steps on the center graph for the hydrolysis of RGR-pNA is shown, with somewhat diminished precision. The KSIE for k_cat_/K_m_ and K_as_ = k_1_/k_−1_ are quite similar, while the KSIE for k_cat_ is clearly a weighted function of k_2_ and k_3_. The substrates have an Arg at the carboxyl side of the bond cleaved and comply with preferences for the P_2_ and P_3_ sides. Both substrates present concave proton inventories under enzyme saturation by them. Again, the best statistical fit is with a model for two equal proton transfers at the TS under saturating concentrations of substrates. The RGR-pNA substrate also gives a small SIE, 0.78, even for k_cat_, indicating a contribution from solvent restructuring, ostensibly associated with rate-limiting leaving group departure. But the fractionation factors calculated at substrate concentrations below K_m_ differ from those for k_cat_.

The KSIEs and fractionation factors for the model best fitting each proton inventory are summarized in Table 2. The KSIEs for k_cat_ are near 3 with simple fluorogenic substrates and 2.2 ± 0.2 for two intramolecularly fluorescence-quenched substrates. Intrinsic isotope effects between 2.5 and 3.5 are most likely primary effects, meaning that protons are transferred in the rate-determining step of the hydrolysis reaction. Proton transfer becomes a component of the reaction coordinate for bond breaking or making at the TS. The fractionation factors for k_cat_ and k_cat_/K_m_ for the rate-determining chemical step in acylation of the enzymes by the substrates containing certain P_1_–P_4_ residues are nearly identical. One or two KSIEs are extracted from the fractionation factors (Table 2) for one or two protons participating in the rate-determining step. The intrinsic KSIEs for proton transfer in the reactions catalyzed by the four enzymes are between 1.0 and 3.9. The fractionation factors are near 1.0 ± 0.4 for solvation terms at saturating concentrations of the substrates except for one. The KSIEs for the k_cat_/K_m_ term are between 1 and 2.1 and present curved proton inventories as all include a term for solvate restructuring. However, the intrinsic KSIEs for the rate-determining proton transfer step are typically near the values obtained for k_cat_.

A deeper insight can be gained from further analyzing the data; an example is for the simplest substrate of thrombin, Z-Pro-Arg-AMC. Its hydrolysis involves a small value of ɸ_s_ for k_cat_ but a larger value for k_cat/_K_m_. The value of 0.8 for solvation in the k_cat_ phase is indicative of some solvent rearrangement accompanying C–O bond formation. Furthermore, the fractionation factor for solvation can be calculated for K_m_ from the ratio of the ɸs as follows; ^V/K^ɸs/^V^ɸs = 1/^K^ɸs = 1.22/0.8 = 1.5, which is the SIE for dissociation: its inverse, the SIE for K_as,_ is 0.67. This is of interest, because K_m_ is an uncomplicated dissociation constant in this case and the SIE simply is a measure of solvate restructuring in the association/dissociation manifold.

At low substrate concentrations, the most reactive thrombin tripeptide substrate due to its optimal sequence, D-Phe-Pip-Arg-pNA, behaves somewhat differently: it has an isotopically insensitive rate-determining step [20,120]. At enzyme saturating concentrations the proton inventory is best fit with the two SHB plus solvation term model, giving a SIE = 0.5 for a solvation term (see Table 2). The acylated analogue of this substrate has been known to be a “sticky” substrate [114], which is entirely commensurate with rate-limiting leaving C–N bond fission and leaving-group departure. This process associated with the highest energy barrier on the reaction path entails a net increase of isotopic fraction factors for numerous solvating proton bridges.

The inclusion of specific P_1_′-P_3_′ residues in the substrates of thrombin introduces more complexity. It elicits large solvent rearrangements at the TS even in the k_cat_ term in one case (FPR-SFR). The decapeptide substrate (Figure 1, right side) shows a domed curve for k_cat_ with a best fit model of two SHBs at the rate-limiting TS sites plus a large fractionation factor for solvation (3.1), which again is consistent with a rate-determining leaving group departure. Nonetheless, the model for two fractionation factors for SHBs at the TS agrees well with the results obtained with tripeptide substrates. However, the thrombin-catalyzed hydrolysis of the nonapeptide substrate (SPR-SFQ) gives a proton inventory with a single proton bridge at the TS, which is in contrast to the short substrates that possess good complementarity at the S_1_–S_3_ subsites and are associated with two-proton bridges at the rate-determining TS. It is as if the introduction of the P’-sites disrupted the correct alignment at P-sites, which introduced compensating terms for intrinsic KSIEs. The existence of exosite interactions in natural substrates may serve exactly to prevent such misfits. The nonapeptide case is consistent with the rate-determining leaving group departure and also with the well known conformational adjustment of thrombin. The lack of binding interactions at the exo site in the reactions of short substrates creates an opportunity to discern the balance between the SHB-assisted chemical steps and physical steps including solvate rearrangements in the reaction sequence and the nature of rate-determining TS under various conditions. The allosteric plasticity of thrombin was investigated further with the natural substrates.

### 5.2. Proton Inventories for the Hydrolysis of Natural Substrates Catalyzed by Thrombin and FXa 

For the first time, three zymogen activation reactions were studied for the elucidation of the role of proton bridges [21,22]. As expected, these reactions posed analytical challenges and the quality of data obtained is inferior to those with substrate mimics.

Thrombin binds in the central region of fibrinogen and cuts off the FpA and FpB peptides in sequence from the Aα and Bβ chains, respectively. Fibrinogen activation to fibrin consists of at least three major steps:

Fibrinogen → FpA + fibrin1 → FpB + protofibril → lateral polymerization → fibrin mesh

**Figure 3:**
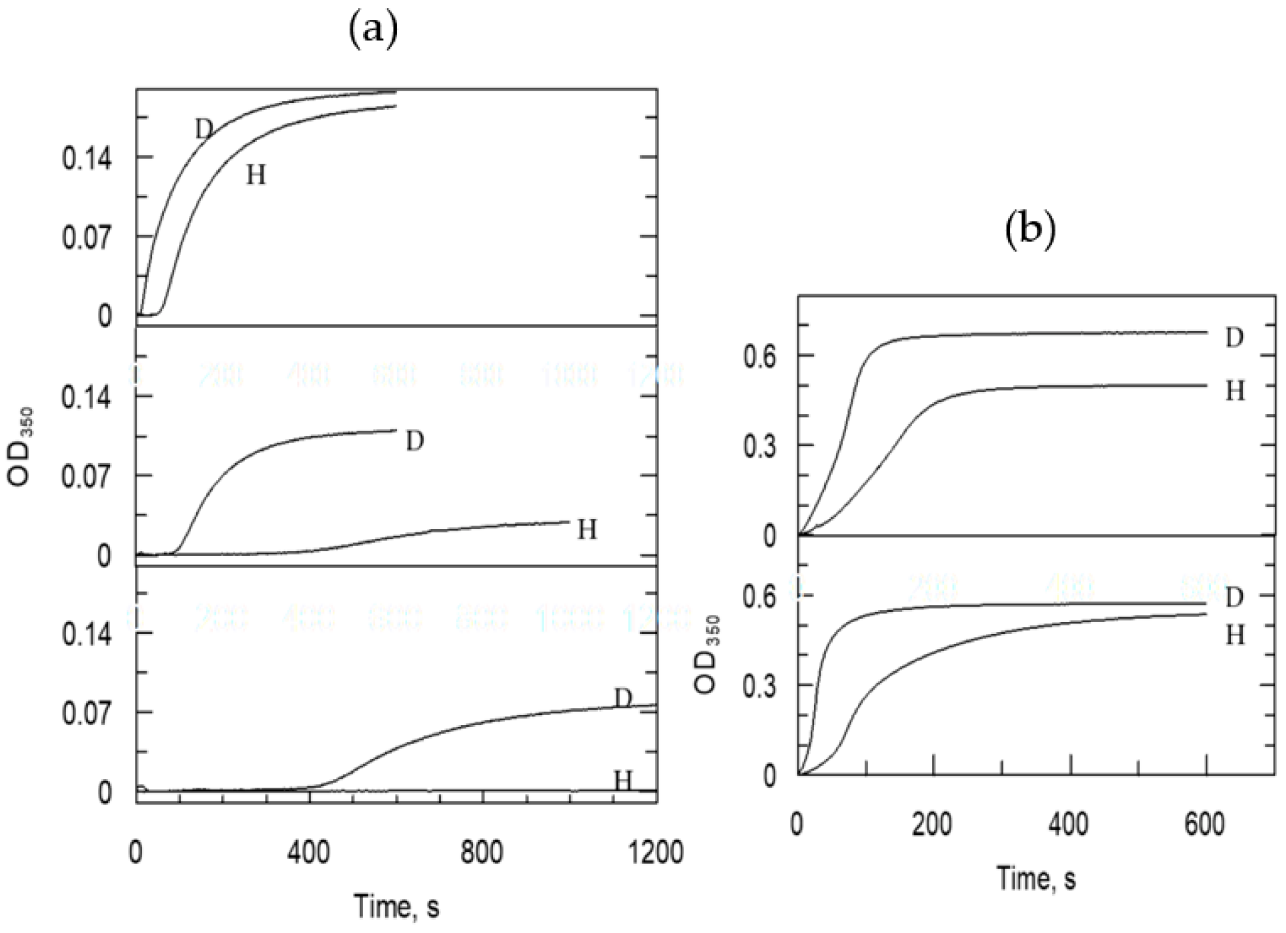
Time course of turbidity development during fibrin formation from 0.33 μM fibrinogen, catalyzed by 1.5 nM thrombin in pH 8.0, 0.02 M Tris buffer containing 0.3 M NaCl, 1% DMSO, 0.1% PEG-4000 at 25.0 ± 0.1 °C, in D_2_O (*n* = 0.88) on the upper curve and in H_2_O on the lower curve and on each graph. (**a**) Left side: in the presence of 0.30 M NaF on top; 0.30 M NaCl middle, and 0.30 M choline Cl, bottom. (**b**) Right side: in the presence of 0.30 M choline Cl and 5 mM CaCl_2_ on top and in 0.30 M NaCl and 5 mM CaCl_2_ on the bottom.

This reaction sequence was studied extensively by monitoring the formation of products and also by analyzing developing turbidity manifest in light scatter. Reports [88,114,116] on turbidity studies claimed that the second bond cleavage and the accompanying lateral polymerization occurring with conformational change are the physical steps monitored in these experiments. The cleavage of the bond to FpA occurs significantly faster than the rest. Accordingly, as shown in Figure 3, the lag time in the curves reports on protofibril formation, then the protofibrils aggregate laterally, which is associated with the maximal slope of the curve. The thickness of the fiber clot may be discerned from the final absorbance. These reactions show a great dependence on specific ions. The top curve on the left side of Figure 3 indicates that in the presence of F^−^ ions, the precipitate is thicker than in the presence of Cl^−^ ions. The thickness of the clot decreases in the presence of cations in the following order; Ca^2+^ > Na^+^ > choline^+^. The presence of a lag time is pronounced in choline Cl^−^ shown in the lowest curve on the left side of Figure 3. Structural effects of ions are more pronounced in H_2_O than in D_2_O. Both the lag time and the maximal slope of the blood clotting curves depend on the ion-perturbed water structure. Structural changes in blood clots are associated with substantial changes in water structure in the direction of looser intermolecular H-bonding accompanying finer particle size. The pivotal importance of the rearrangement of water channels during conformational changes in FpB formation is consistent with this unique observation.

**Figure 4:**
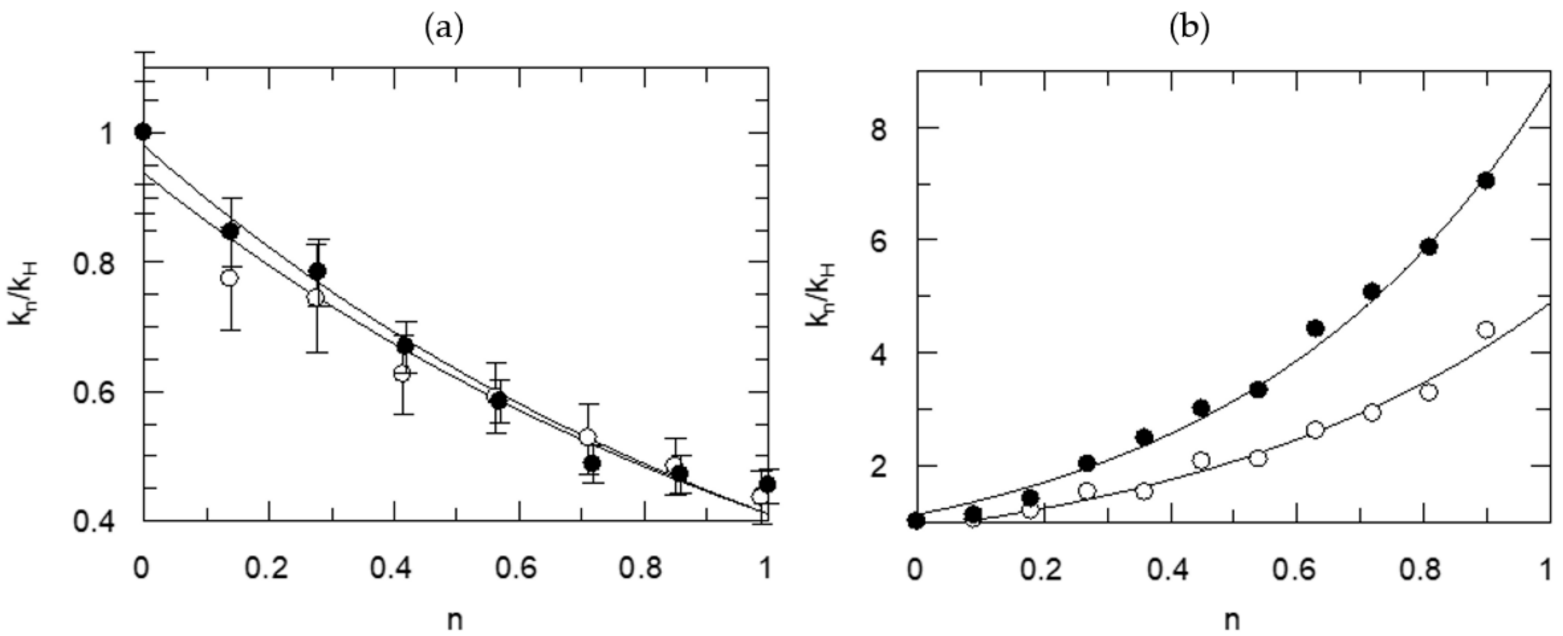
Proton inventory for the thrombin-catalyzed fibrinogen hydrolysis at conditions indicated in Figure 3. (**a**) Left side: open circles, initial rates at 22 μM fibrinogen and the equation of the line is k_n_/k_H_ = 0.90 (1 − n + n/1.51)^2^; filled circles, pseudo-first-order data at 0.55 μM fibrinogen and the equation of the line is k_n_/k_H_ = 0.98 (1 − n + n/1.56)^2^. (**b**) Right side: proton inventory for the evolution of turbidity during fibrin formation. Open circles represent inverse partial solvent isotope effects for the inverse lag (life) time and the line was calculated from τ_n_/τ_H_ = 0.88 (±0.06) [5.55(±0.52)]^n^ Closed circles are for the maximal slopes of turbidity plots and the line was calculated from slope_n_/slope_H_, a = 1.13 (±0.06)[7.79 (±0.56)]^n^ Errors are within the symbols.

A systematic proton inventory study, in the presence of NaCl, of the lag times and slopes of the blood-clotting curves, shows exponential dependence on the atom fraction of D in the buffer displayed in Figure 4 on the right side. Consequently, the KSIEs are very inverse and, thus, the fractionation factors are large for solvent rearrangement, probably, when FpB forms. The equations of the lines are derived from the best-fit all-solvation model, which depicts a conglomerate of small inverse SIEs each from a solvation site in the polymerization process. The SIEs are more inverse in the presence of Na^+^ and Cl^−^ ions that promote lighter clotting, indicating a net decrease in the strength in H-bonding. The introduction of D in the buffer counteracts this trend. As substrate concentrations were enhanced, the SIEs became increasingly inverse. This result supports that with the tightening of interlocking H-bonds, their strength amplify and/or the water structure tightens around the lateral fiber. The phenomena of inverse KSIEs are corroborated by other polymerization reactions that involve major solvent restructuring.

Figure 4 also shows on the left side, the proton inventory for fibrinogen activation monitored by HPLC. The Na^+^ ion concentrations were varied to probe the catalytic machinery as it adopts the efficient conformation of thrombin operating in fibrinogen activation. Remarkably, the statistically most relevant model of the proton inventory curves for k_cat_ and k_cat_/K_m_ indicated the participation of two similar (identical) short proton bridges with intrinsic fractionation factors 0.64–0.66 in fibrinogen processing by thrombin. Most likely, it is associated with the first covalent step in the natural reaction, i.e., the acylation of the catalytic Ser during bond cleavage to FbA. The bond forming/breaking is aided with a concerted dual proton transfer to bring down the TS potential energy barrier. Solvent restructuring does not show a SIE when the two-proton model is calculated. Thus, the proton inventory of this acylation reaction is uncomplicated by comparison to the internally fluorescence-quenched peptide substrates of thrombin, vide supra. Similarity appears between proton inventories for fibrinogen and the p-NA peptides that give similar TS fractionation factors for the two-proton bridge model (Table 2). The internally fluorescence-quenched decapeptide mimic of FVIIIa also shows two and similar TS fractionation factors. An accompanying large-inverse fractionation factor indicative of a substantial solvent rearrangement (Figure 1) is consistent with rate-limiting leaving group departure. This is not the case for the natural substrate, which may receive aid for leaving group departure from remote-site interactions. Amide bond cleavage may be partly or fully rate determining in fibrinogen hydrolysis at substrate concentrations both below and above saturating the enzyme. Two other methods exist to untangle the difficulty of masked chemical steps; the variation of temperature of the reaction or the electrostatic milieu can be changed. Di Cera’s group [114] applied this technique to fibrinogen activation and found that the TS energy barrier originates 67% from the acylation step and 33% from the encounter step at 37 °C but under different experimental conditions to those employed in this study [21].

**Figure 5:**
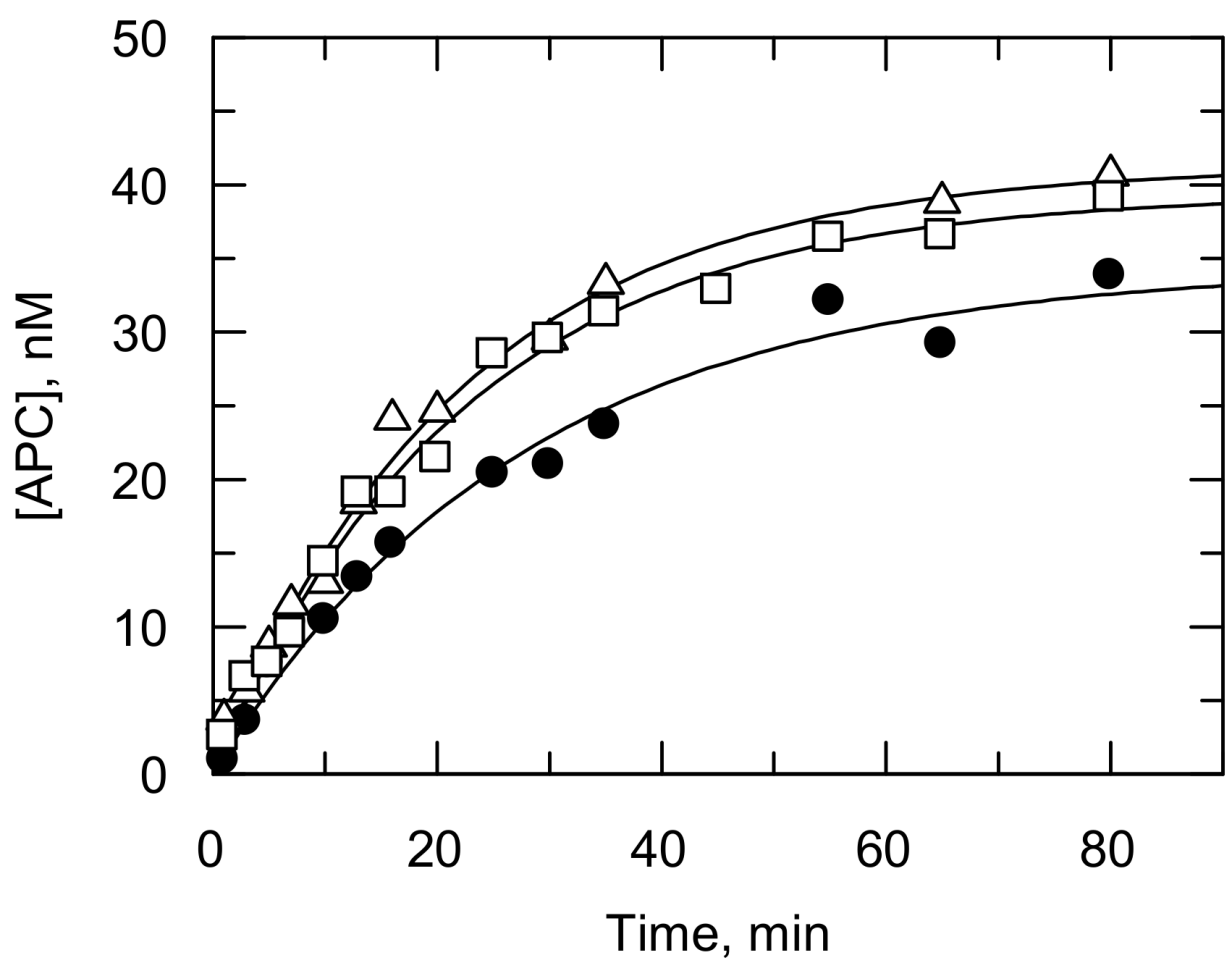
Solvent isotope effect for thrombin-catalyzed PC activation at pH 8.5, 0.02 M Tris, 0.30 M NaCl, 5 mM CaCl_2_, 0.1% PEG-4000, 100 nM TM, 13.1 nM thrombin and 37.0 ± 0.1 °C. Circles represent H_2_O buffer, triangles D_2_O buffer (*n* = 0.91) and squares H_2_O/D_2_O buffer (*n* = 0.45). The errors are smaller than the symbols.

**Figure 6:**
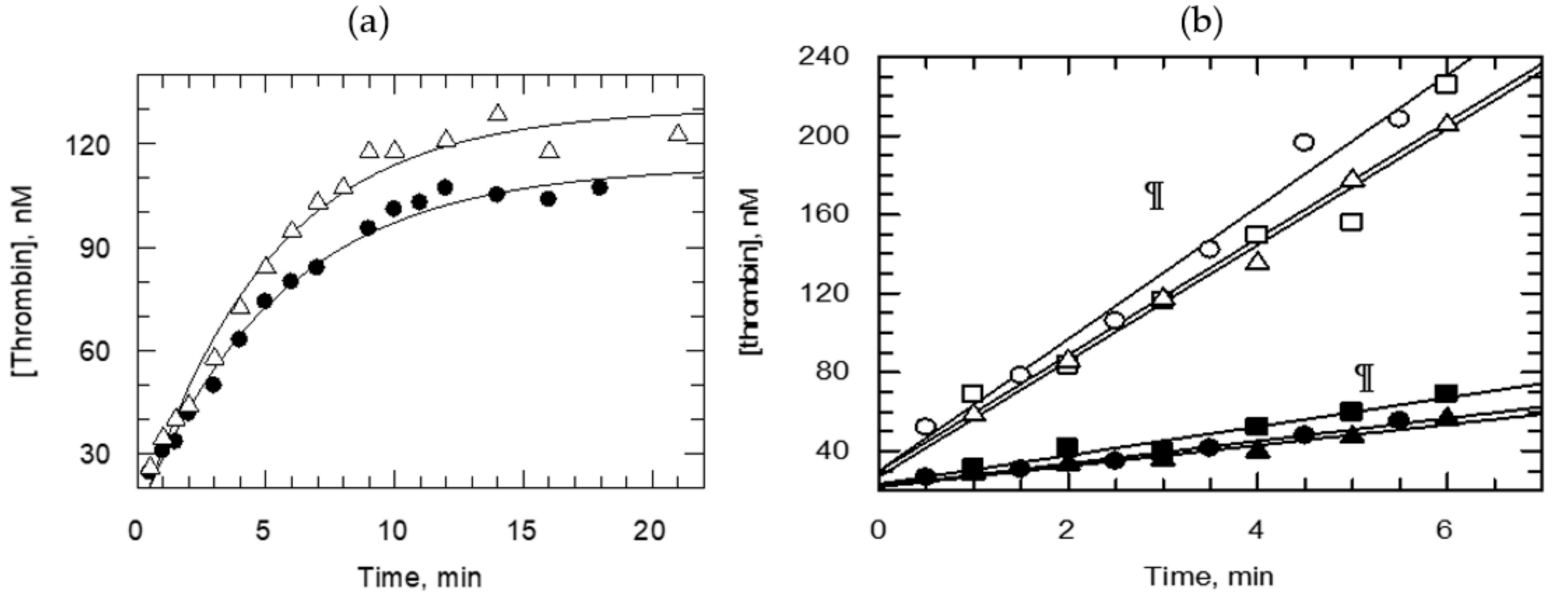
Human FXa-catalyzed human prothrombin activation in the presence of four-fold excess of FVa, at pH 7.50, 0.020 M Tris buffer, 5.0 × 10^−5^ M LUV, 5 × 10^−3^ M CaCl_2_ and 25.0 ± 0.1 °C. (**a**) Left side: Time dependence of the reactions, the filled circles represent H_2_O buffer and triangles D_2_O buffer. (**b**) Right side: Initial rates, the filled symbols represent H_2_O buffer and open symbols D_2_O buffer.

Zymogen activation catalyzed by thrombin to form APC from PC and the FXa-catalyzed activation of prothrombin in the presence of FVa, phosphotidylcholine and phosphatidylserine (75:25) was studied at low substrate concentrations. All reactions were associated with small inverse SIEs, illustrated on Figure 5 and Figure 6. Apparently, bond fission in these reactions is not the rate-determining step under the conditions studied. Activation of PC to APC by thrombin was monitored using a coupled assay for increasing APC activity with the aid of an APC-specific chromogenic substrate [87,118]. There are fluorogenic substrates that provide greater selectivity for APC [117]. However, the safest technique for the proton inventory measurements is quenching PC activation in the appropriate solvent medium by inactivating thrombin with antithrombin III or hirudin and assaying for emerging APC activity in water to exclude any contribution of the assay reaction to solvent isotope effects. The rate of the reaction dropped to half in the absence of Na^+^ ions and a lag time appeared. Otherwise, this process is known [88] to be catalyzed by the “slow” form of thrombin, which is less dependent on Na^+^ ions. The inverse effect in D_2_O is amplified in the presence of Na^+^ and Cl^−^ ions as the SIE drops from 1.02 ± 0.06 to 0.75 ± 0.09 when 0.30 M choline chloride replaces 0.30 M NaCl. Although the reaction mechanism of PC activation is not well known, the inverse SIE indicates a rate-limiting physical step in this reaction.

Prothrombin activation by FXa starts with association followed by rearrangement at the aqueous–lipid interface, which are likely to limit the rate. The very role of activation is fulfilled by juxtaposing the reactants on heterogeneous surfaces that enables the chemical transformation. Thus, strong contributions are expected from solvent rearrangement in the FXa-catalyzed physiological reaction. Indeed, very inverse SIEs, 0.2–0.3, were calculated from the data obtained (Figure 6) in the presence of FXa:FVa in 1:4 ratio and 50 μM phospholipid vesicles (LUV), while the substrate concentrations were raised above K_m_. The implication is that physical rather than chemical steps dominate the rate-limiting phase of prothrombin transformation to thrombin at the aqueous–lipid interface. In fact, the SIE becomes increasingly inverse, reaching a value of 0.19 at high prothrombin concentrations. Meizothrombin intermediate is formed under these conditions but it is quickly channeled to thrombin without release from the membrane-enzyme complex [104]. A conformational switch is required in this case to poise the Arg^322^-Ile^323^ bond for fission. This optimal projection of the proteolytic site of prothrombin is supported by binding to the exosite of FXa and near the carboxyl segment of the FVa heavy chain. Clearly, this requires a grand reorganization of myriads of water solvates, which contribute to fractionation factors between 3.3 and 5. Thus, the proton inventory results are in full accord with this physical event being rate limiting. In the presence of FXa:FVa = 1:1 and at enzyme saturation with substrate, the SIEs become small normal, i.e., greater than 1.0. A reversal of the order of bond fission in prothrombin is likely under these conditions as FVa concentrations are low. This has been the case in the absence of FVa in the reaction mixture [104]. As shown in Figure 2, an unmasked picture of the rate-determining events in peptide bond hydrolysis catalyzed by FXa, studied with natural substrate mimics in aqueous buffer, presents very different proton inventories. FXa-catalyzed reactions of chromogenic substrates behave similarly to the thrombin-catalyzed reactions of oligopeptide p-NAs or 7-AMC, i.e., show two-proton bridges at the rate-determining TS. However, N-α-Z-D-Arg-Gly-Arg-pNA·2HCl, a very efficient substrate of FXa, hydrolyzes with a contribution also from solvent rearrangement at the rate-determining TS while saturating the enzyme.

### 5.3. Probing the Proton Bridges in Covalent and Non-Covalent Adducts of Thrombin

**Figure 7:**
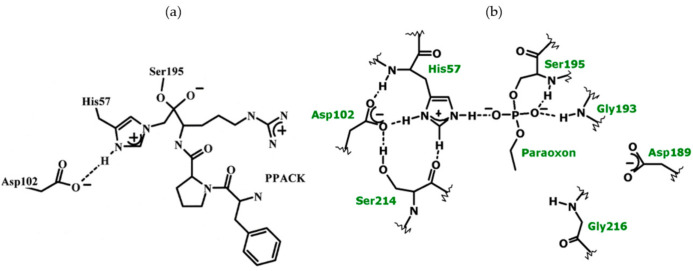
(**a**) Left side: structure of Phe-Pro-Arg-chloromethylketone (PPACK) covalently linked to the catalytic triad of thrombin. (**b**) Right side: structure of paraoxon-inhibited thrombin after dealkylation (modified structures of the thrombin active site from the Protein Data Bank entry ISHH).

Mechanism-based inhibitors have been used broadly as investigative tools for the elucidation of fleeting TS structures not amenable to immediate observation. A truly effective affinity label of thrombin is Phe-Pro-Arg-chloromethylketone (PPACK) as it consists of amino acids that fulfill the requirements for the P_1_-P_3_ binding subsites. It attaches to the active site Ser^195^ and crosslinks with His^57^, thus creating a good mimic of the tetrahedral intermediate in the acylation of thrombin, shown in Figure 7 on the left side [23]. The correspondence between amino acids in the PPACK and subsites at the thrombin active site enforces tight interactions. The proton bridges of the catalytic apparatus may thus be compressed. Another broadly tested group of covalent modifiers of serine hydrolase active sites are phosphate and phosphonate esters. The negative charge density accumulates around the esters, which repels nucleophilic attack at P for either self-catalyzed or chemical reactivations. Frequently, this is further exacerbated if an alkoxy ligand hydrolyzes off or dealkylates. Dealkylation also occurred when thrombin was inactivated with paraoxon and NPMP (4-nitrophenyl-2-propyl methylphosphonate). Figure 7 on the right side shows the dealkylated product at the paraoxon –inhibited thrombin active site. The charge density is thus further enhanced and it and the geometry of this structure resemble the intermediates in deacylation.

#### 5.3.1. Kinetic Characterization of Thrombin Inhibition with Covalent Modifiers

Inhibition reactions were carried out [23] as a function of concentration of [I], pH, temperature and ionic strength. The data analysis was performed in accord with Equation (2), as the rate constants showed hyperbolic dependence on [I] consistent with Michaelis Menten kinetics. First-order rate constants were calculated from data obtained at [I] << K_i_ and second-order rate constants were calculated from inhibitor dependence of the observed rate constants. From the dependence on PPACK concentration, the individual rate constants were also calculated although with low precision. The kinetic and thermodynamic data are tabulated in Table 3.
K_i_^−1       ^k_h_/k_−h_       k_alk_ E + I 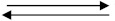 EI 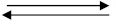 EI* 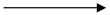
EI**(2)

The kinetics of binding of small inhibitors resemble the initial steps of reactions of normal substrates. As discussed earlier, the inhibition of thrombin with PPACK occurs with the intervention of a tetrahedral covalent adduct which forms without the departure of any fragment from the molecule and thus resembles the tetrahedral intermediate formed after nucleophilic attack on a substrate of thrombin. The maximal second-order rate constant, k_i_/K_i_, for the inhibition of human α-thrombin with PPACK was determined to be 2.15 × 10^7^ M^−1^s^−1^ at pH 8.1 and 1.07 × 10^7^ M^−1^s^−1^ at pH 7.00 and 25.0 ± 0.1 °C. The K_i_ value for PPACK inhibition of thrombin is over 100 fold smaller than the K_m_ value for the best chromogenic substrate of thrombin, FPiR-pNA, at pH 8.0 [20,113,118]. The tripeptide segment of the two structures are nearly identical as Pip is an analog of Pro. Although K_m_ is a complicated constant with contributions from rate constants for elementary chemical steps, its numerical value is identical to the dissociation constant for the reaction of FPiR-pNA with thrombin. The implication is then that K_i_ for thrombin inhibition by PPACK is reduced by a factor which includes terms pertaining to events ensuing the binding step (Equation (2). It is almost certain that formation of the hemiketal anion, k_h_, precedes alkylation of His^57^ by the methylene group and the departure of Cl^−^ (k_alk_). It probably contributes to the rate-determining step in Equation (2). The kinetic and thermodynamic data of covalent inhibition of thrombin are in Table 3.

The activation barrier for the self-catalyzed inhibition of thrombin by PPACK is given by the values of ΔH^‡^ = 10.6 ± 0.7 kcal/mol and ΔS^‡^ = 9 ± 2 cal/mol K. The activation free energy of ΔG^‡^ = 7.85 kcal/mol can be calculated from the second-order rate constant at pH 7.00 and 25.0 ± 0.1 °C, calculated using the Eyring equation. The difference between ΔG^‡^ and ΔH^‡^ is 2753 cal/mol, the value of TΔS^‡^, which gives ΔS^‡^ = 9 cal/mol K. A nonlinear fit of the temperature dependence of the second-order rate constant gives a value in great agreement with this value. This means that the TS of the rate-determining step for k_i_/K_i_ has more favorable entropy than the free enzyme and 1 M inhibitor have. Thus, the TS has a more favorable electrostatic environment associated with formation of the hemiketal anion than the TS of a simple noncovalent association of the enzyme and the inhibitor. By comparison, the thrombin-catalyzed hydrolysis of FPiR-pNA, the structural substrate analog of PPACK, gave a value of ΔH^‡^ between 10.21 and 11.14 kcal/mol for the acylation rate constant (k_2_) under similar conditions [115,118]. The TSs for the two reactions have similar quasi-tetrahedral character and similar charge distribution.

From Table 3, the pK_a_ of 7.3 is consistent with catalysis by His^57^ as the unprotonated His base is needed to promote the reaction. The pK_a_ of the amino terminal Ile^16^ residue, which is engaged in a salt bridge with Asp^194^ thereby keeping the oxyanion hole in the correct conformation for catalysis, agrees well with a second pK_a_ of 8.8. This observation has also been reported for other thrombin-catalyzed reactions, including those in this study [20,23,118,120].

Another intermediate occurs in the substrate reaction sequence in the course of deacylation after water attack on the acyl enzyme. Phosphate and phosphonate ester adducts of thrombin especially after dealkylation (aging) model this intermediate [23,27,28,94,96,140,141,142,143,144,145,146]. Deacylation in a natural reaction of thrombin involves significant negative charge accumulation at the oxygens of the tetrahedral intermediate, which also occurs at P in the in phosphorylated active sites [146]. However, paraoxon and NPMP inhibit thrombin 7 and 6 orders of magnitude less efficiently than does PPACK. The pH-rate profiles of the two classes of inhibitors are quite different. The pK_a_ of His^57^ in the phosphate and phosphonate adducts approaches 8.0, which is consistent with the enhanced negative charge accumulation described. Whereas these pK_a_s are half a unit higher than that for PPACK inhibition, the upper pK_a_ value assigned to the more remote Ile^16^, are similar in the three adducts because the stronger negative electrostatic environment around P enhances the basicity of His^57^ only.

The KSIEs are near unity for the second-order rate constants of all three inhibition reactions of thrombin. This result is not surprising as the k_cat_/K_m_ for the thrombin-catalyzed hydrolysis of FPiR-pNA is also associated with a unit KSIE. The fractionation factor for the TS of the association of thrombin with FPiR-pNA or that of the sequential conformational change is one [20]. The fractionation factors for the encounter between thrombin and PPACK is 1.0 at pH 7.45 and 0.93 at pH 8.00 within ~10% experimental error. These results with thrombin deviate from the solvent isotope effect obtained for elastase inhibition by a peptidyl chloromethyl ketone, which is 0.65 at [I] < K_i_ [64]. Interpretations of this result, however, include the possibility of a TS fractionation factor of 1.0 for k_i_/K_i_, if there is a compensation of terms due to the contribution of solvent reorganization in elastase inhibition by the peptidyl chloromethyl ketone. Ostensibly, the significant differences in the subsite binding environments of the two enzymes and the allosteric flexibility of thrombin lie at the heart of the differences.

#### 5.3.2. SHB in Covalent Adducts Detected by Low-Field ^1^H NMR

The low-field ^1^H NMR signals at 18.10 and 17.34 ppm for the analogs of the tetrahedral intermediate for acylation and of the intermediate for deacylation, respectively, establish the presence of an SHB in the covalent adducts of thrombin [23]. PPACK-inhibited thrombin shows a peak at 18.10 ± 0.05 ppm in 7% D_2_O and at 17.8 ppm in 55% D_2_O, while the peak for the integration standard proton sponge in acetonitrile-d_3_ is positioned at 18.60 ± 0.05 ppm. This result was reproduced several times. The line width of the signal at 18.10 ppm sharpened with increasing temperature relative to the proton sponge, indicating a faster proton exchange at the 18.10 resonance. The ^1^H NMR signal with paraoxon-inhibited thrombin at 17.34 ppm in 7% D_2_O was assigned to the dealkylated adduct as it emerged slowly and reached a maximum after 50 h. After the loss of the ethyl group, strong negative charge accumulates on the oxygen at P and provides deshielding of the proton bridge. Nearly identical results were obtained with the phosphonate inhibitor, NPMP. The highly deshielded signals occurring in these adducts reflect the negatively charged environment deeply buried in the canyon where the active site resides.

From the low-field signal intensity measured in different isotopic mixtures of buffered water, an isotope effect of 2.2 ± 0.2 was calculated for the formation of a proton bridge which occurs at the active site in the PPACK-inhibited thrombin. Again, this isotope effect denotes the presence of an SHB. The minima in the vibrational potential energy well for the H-bond, vide supra, are typically separated by distances between 0.4 and 0.7 Å [74]. An empirical correlation can be derived, a third-order polynomial fit of ɸ to the distances between the minima of the two vibrational potential energy wells [7,10,11,14,23,24,25,26,68,69,70,71,72,73,74,79,80,81]. Because the covalent bond length in O-H and N-H bonds are close to 1.00 Å, two covalent bond length, 2.00 Å, are added to the distance between the minima of the wells to yield the distance between donor and acceptor in a proton bridge. Performing this calculation yielded the same bond length of 2.62 Å and with the same precision as estimated from the chemical shifts for the PPACK- inhibited thrombin using a correlation of chemical shifts and N–H–O bond distances in small crystals [14,23,24,25,26]. Donor-acceptor distances of His^57^δNH and Asp^102^γO or Asp^102^γO and Ser^214^γOH in PPACK-inhibited thrombin from crystallographic data compare well with these finds [87]. Adducts of paraoxon and NPMP with thrombin gave similar values, 2.62–2.64 Å, to that calculated for the SHB in the adduct with PPACK.

Notably, in corresponding analogs of tetrahedral intermediates in serine proteases [7,10,68,69,70,71,72,73,74] and in the double-displacement mechanism of ester hydrolysis catalyzed by cholinesterases [14,24,25] nearly identical resonances were reported to the three cases with thrombin. The protonated His^57^ of the native enzymes gave a similar peak in most cases at pH below 6. In contrast, we have not found a signal with the native thrombin between pH 5.3 and 8.5. While proton exchange is faster in thrombin than in the serine hydrolases interrogated [92], the proton bridge at the active site in the native thrombin appears to be longer than the proton bridges at the active site of other serine proteases. This may be due to the vicinity of residues around the proton bridge in the His-Asp pair, which can serve as catalysts of proton transfer in a relay at the pH values in our study.

As the kinetic studies demonstrated (Table 3), the TS for binding the small inhibitors does not manifest in SHBs, but the stable adducts of the same inhibitors with thrombin show at least one unique SHB at the active site. This is in agreement with that found for the first step of hydrolysis of fibrinogen to FpA, shown on the left side of Figure 4 [21]. It appears reasonable to assume that these SHBs also occur in tetrahedral intermediates on the thrombin-catalyzed reaction path. However, these small-molecule modifiers of the thrombin active site lack critical remote interactions of natural substrates at exosites. In particular, the fibrinogen binding exosites determine the substrate selection regulated by Na^+^ ion binding at an adjacent location [87,88] as delineated in Section 4.1.

#### 5.3.3. A 1 ns Molecular Dynamics (MD) Simulation and Preliminary Quantum Mechanical/Molecular Mechanical (QM/MM) Calculation 

Quantum mechanical/molecular mechanical (QM/MM) and MD calculations targeted all systems under high-resolution ^1^H NMR investigations [147]. The conformational mobility of the active-site region in thrombin was examined first in the MD simulation. This simulation indicated a tight H-bonding arrangement between Asp^102^ and His^57^ being part of the catalytic mechanism of thrombin. The His^57^ Nδ-H-O Asp^102^ distance is 2.8 Å in native thrombin [103,147], 2.71 Å in the D-Phe-Pro-Arg chloromethylketone adduct [101,102] and ranges between 2.7 and 2.8 Å in a phenacyl methylphosphonate adducts of thrombin [27].

**Figure 8:**
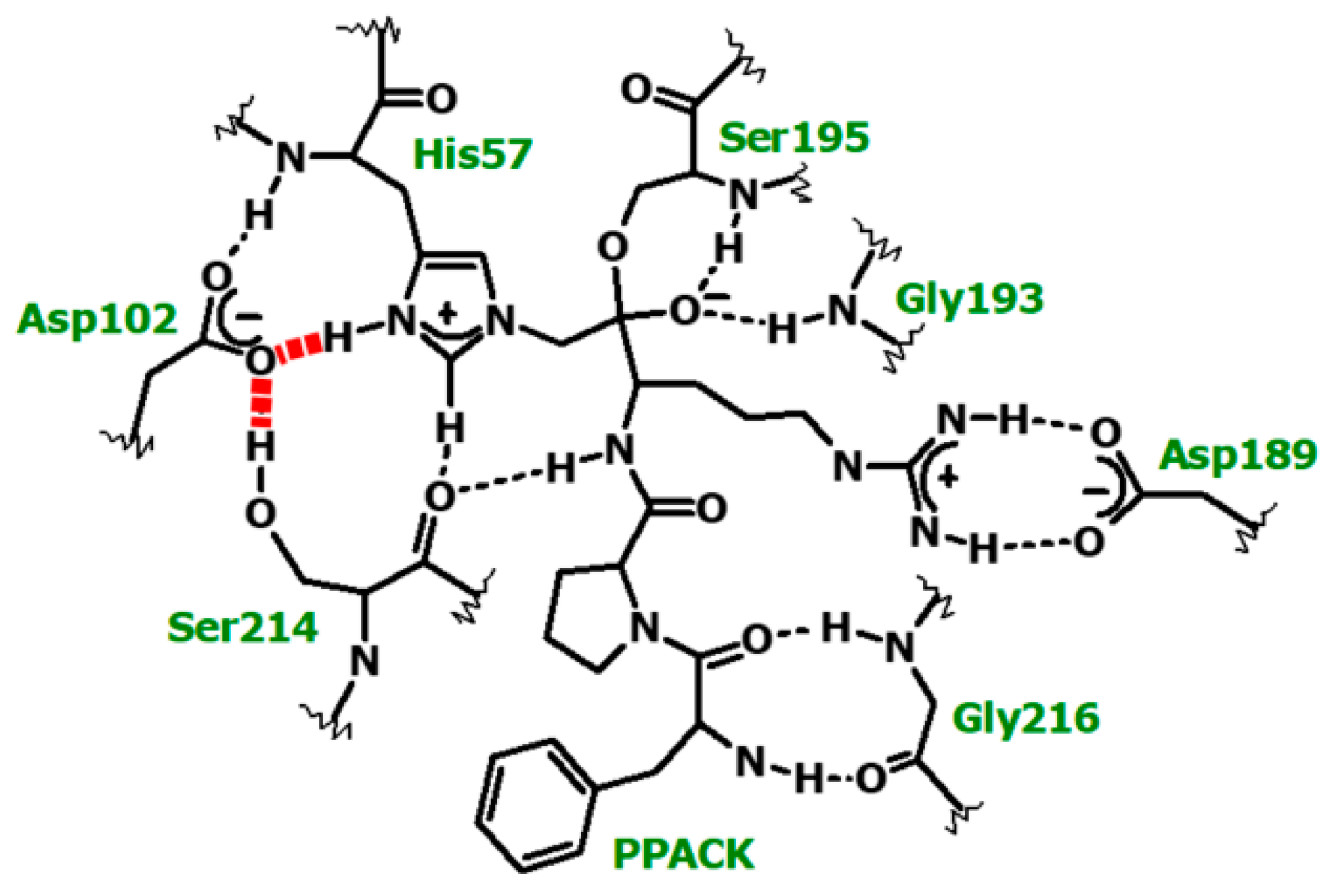
α-Thrombin active site inhibited with PPACK.

A new question emerged from the MD simulations of the PPACK-inhibited thrombin; it showed two competing potential SHBs at the active site of thrombin. One is an SHB between His^57^ Nδ and Asp^102^ Oβ originally proposed [6,148] and the other is one between Ser^214^ Oγ and Asp^102^ Oβ, an equally likely candidate (Figure 8). The MD approach used for the α-thrombin-PPACK covalent adduct showed equal frequency of the occurrence of the two H-bonding options. A more refined calculation uses QM/MM at the B3LYP/6-31G** or RHF/6-31G(d) level of theory. The results of the completed one ns CHARMM [93] simulation of this system are the starting structure for a CHARMM-Qchem calculation. There are about 45 core atoms at the active site. Figure 8 shows the core of the assembly with the two potential SHBs. Both cases indicate ~150 ps periods for SHB formation at donor acceptor distances <2.6 Å for either donor-acceptor pair, during a ns simulation. A refinement of these results is what the QM/MM calculations target. The results of this brief calculation so far point to a slight preference for the His^57^-Asp^102^ pair.

It is interesting that in a quantum chemical calculation for RNase A catalysis, contraction of a proton bridge indeed facilitated nucleophilic attack on C. The activation barrier for this reaction was reduced by an SHB in the catalytic apparatus [149].

#### 5.3.4. SHBs in Non-Covalent Adducts of Thrombin

Whether or not SHBs have a role in tight-binding interactions between thrombin and hirudin, as it exists between thrombin and cognate substrates, was investigated next [26]. First, hirudin forms 13 intramolecular H-bonds in its tight core, yet a solution of hirudin alone does not resonate below 12 ppm in ^1^H NMR. Hirudin binds to thrombin with a K_i_ of 20 fM. Tight binding of hirudin showed strongly ion-dependent KSIEs around unity in kinetic and equilibrium experiments [150]. But the X-ray structure of the complex also shows numerous intermolecular proton bridges interspersed with salt bridges near the FRS exosite. As discussed, this site is crucial in the allosteric regulation of thrombin function. This is supported by 2D ^1^H NMR studies of r-hirudin bound to and without thrombin, which revealed that the C-terminal section of hirudin lost some order in solution relative to the same 2D ^1^H NMR result with hirudin in solution alone [51,151,152,153].

**Figure 9:**
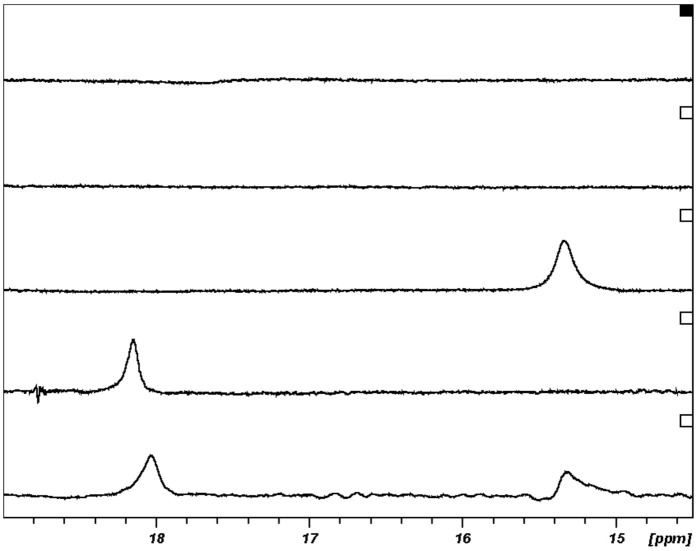
Low-field sections of 3000 transients of 600 MHz ^1^H NMR spectra of human α-thrombin and its inhibited forms at pH 6.5, 0.05 M citrate buffer, 0.20 M NaCl, 0.01% PEG800, 30.0 ± 0.1 °C and 7% D_2_O: human α-thrombin; r-hirudin in 0.01 M phosphate buffer; r-hirudin-inhibited thrombin; PPACK-inhibited thrombin; and PPACK added to the thrombin-r hirudin complex; from top to bottom, respectively. The line width at half height is 60 Hz for the thrombin-PPACK adduct and 98 Hz for the thrombin–hirudin complex.

The characterization by low-field ^1^H NMR, of SHBs in thrombin when inhibited with the non-canonical r-hirudin, its mimics and in comparison to canonical peptide inhibitors posed challenges. We had previously recorded the ^1^H NMR spectra for thrombin and PPACK-inhibited thrombin shown in Figure 9. A signal at 15.33 ppm arose upon addition of r-hirudin to a thrombin solution, while r-hirudin gave no perceptible signal by itself and no new signal could be detected when r-hirudin was added to the solution of thrombin-PPACK adduct (Figure 9). The experiments gave identical results whether type 1 or type 2 hirudin was used. Both signals, at 18.10 ppm and 15.33 ppm, meet the requirements for an SHB, but the one observable with PPACK at the active site is in an environment of lower electron density than the SHB formed between thrombin and hirudin or its mimics. Notably, when PPACK was added to the thrombin-hirudin complex, the signal for the thrombin-PPACK adduct moved upfield by 0.1 ppm and the peak at 15.33 ppm moved downfield by <0.1 ppm in the ternary complex. Both peaks broadened and became somewhat skewed, which may indicate a proton exchange between the active site and the interface between thrombin and hirudin. PPACK seems to be somewhat less tightly bound to the active site in the ternary complex. The signals are pH independent between 5.6 and 8.8. The proton exchange rate constants at pH 6.5 were calculated at 60 Hz for the PPACK adduct and at 98 Hz for the hirudin complex each at half peak height. The corresponding rate constants are 188 s^−1^ and 307 s^−1^ at 30 °C for the thrombin-PPACK adduct and the thrombin-hirudin complex, respectively, unless dipolar effects also contribute to the line width. Polarization effects resulted in peak broadening at 5 °C. Frey and coworkers reported a similar observation [70].

**Figure 10:**
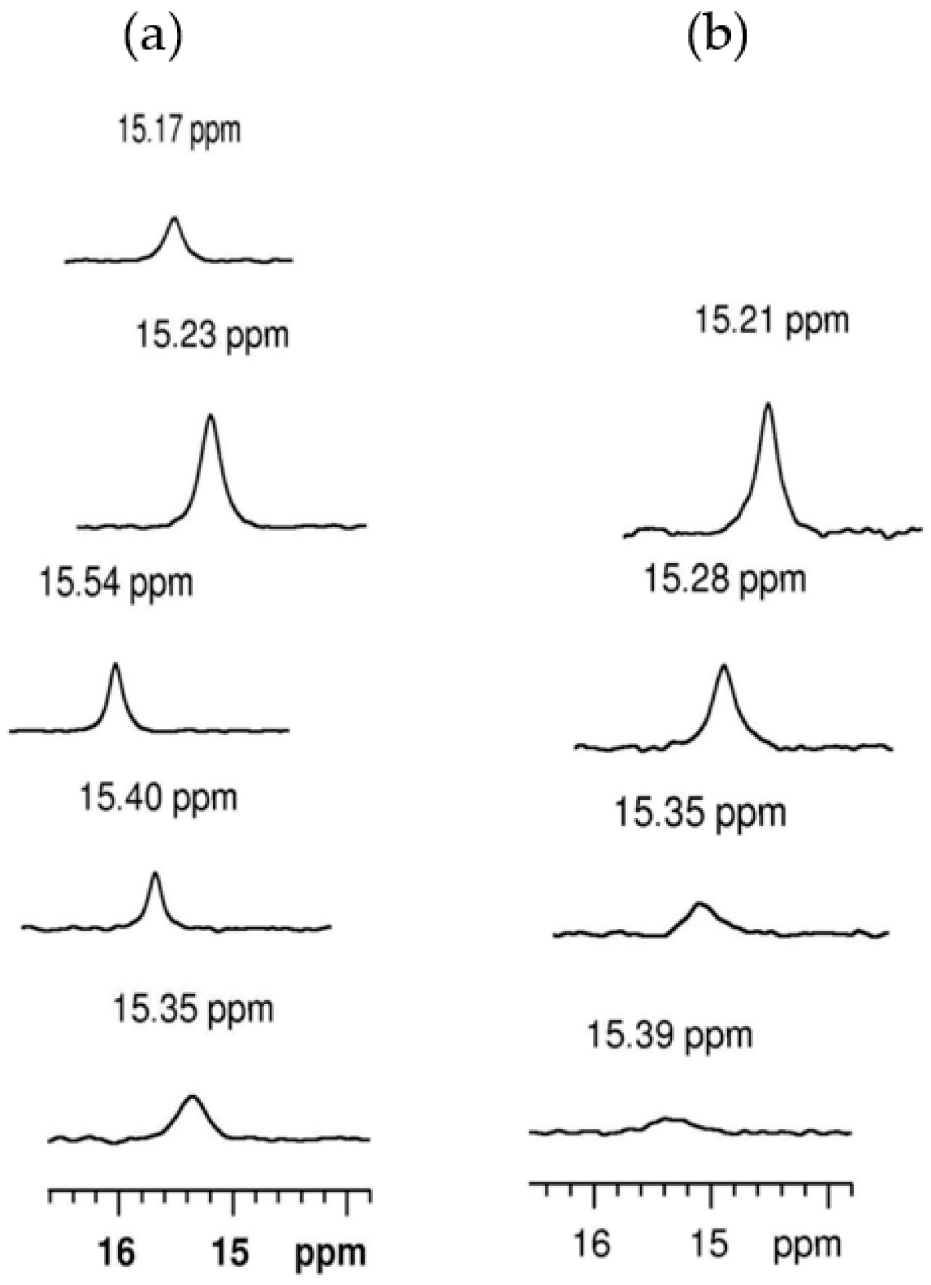
(**a**) Left side: Low-field region of the spectra (3–4000 transients) obtained for complexes of human α-thrombin with hirunorm V, hirunorm IV, RGD-hirudin (recombinant 32SGD34 type 2 hirudin), Nα(Me)Arg-peptide and D-NAPAP (Nα-2-naphthylsulfonyl-glycyl-DL-4-amidinophenylalanyl-piperidide acetate) from top to bottom, respectively, in pH 6.5, 0.025–0.050 M citrate buffer, 0.10–0.20 M NaCl, 0.005–0.010 % PEG-8000 at 30 °C. The corresponding peak widths at half height are 91, 82, 43, 38 and 125 Hz. (**b**) Right side: Temperature dependence of the low-field resonance for the human α-thrombin-hirunorm IV complex in pH 6.5, 0.020 M citrate buffer, 0.10 M NaCl and 0.005% PEG8000. The spectra were recorded at 35, 25, 15, and 5 °C, from top to bottom.

The resonances of the adducts of thrombin with hirudin mimics were similar to that of the thrombin hirudin adduct, displayed on the left side in Figure 10. Further experiments with the hirunorm V-inhibited thrombin in the temperature range 5–35 °C gave the peaks on the right side of Figure 10. Peak broadening and a shift to lower field with decreasing temperature are seen, which may be attributed to decreasing solubility and aggregation of the adduct or polarization effects. The effect of Na^+^ ion concentration on the resonance obtained with thrombin complexed to hirudin and its analogs was not perceptible in the range on 0.09–0.31 M.

The ^1^H NMR resonance obtained with the t-RGD-hirudin is 0.21 ppm more deshielded than the hirudin signal which may be due to changes in the environment of the interactions between the FRS, a critical binding region, and the RGD sequence. RGD replaces an SDG sequence in the natural hirudin. In contrast to the RGD-hirudin signal, the complex of the Nα(Me)Arg-peptide with thrombin gives essentially the same resonance as the thrombin-r-hirudin complex does implying that the same or similar binding elements are involved in the two cases. The Nα(Me)Arg-peptide was designed for enhanced binding affinity and storage stability by introducing methylation of the Arg in the P_1_ position to exclude binding at this site [52,154]. This close analog of the C-terminal peptide of hirudin is tethered to the N-terminal segment with a poly-Gly linker, which interacts with human α-thrombin (K_i_ = 37 pM).

The complex between thrombin and a canonical inhibitor, D-NAPAP (Nα-2-naphthylsulfonyl-glycyl-DL-4-amidinophenylalanyl-piperidide acetate), shows a weak signal at 15.35 ppm with a line width of 125 Hz broader than in the other complexes (Figure 10). D-NAPAP binds at the binding region of the active site in thrombin in the predominant canonical orientation as is the case with PPACK [155]. D-NAPAP also inhibits trypsin and binds in a similar manner at the active site, yet it does not give a signal at lower field than 12 ppm from DSS (4,4-dimethyl-4-silapentane-1-sulfonic acid sodium salt) under the conditions of our experiments. The canonical inhibitors form an antiparallel β-sheet with the Ser^214^-Gly^216^ segment of thrombin. Twin H-bonds are prevalent between the backbone N and O of Gly^216^ and the O and N of Gly in the P_2_ position of D-NAPAP [155,156], as they occur in the PPACK-bound thrombin. The peak at 15.35 ppm indicates the presence of an SHB probably in this region. If D-NAPAP binding results in sequestering or compressing the active site of thrombin, an SHB may form in the catalytic triad. While His^57^ in the thrombin-D-NAPAP complex is likely to be protonated at pH 6.5 to give rise to the signal, this contradicts the absence of the low-field ^1^H NMR resonance in native thrombin at this pH. The occurrence of an intermolecular SHB at a binding site is just as possible. By comparison, chymotrypsin forms a neutral hemiacetal with *N*-acetyl-l-leucyl-l-phenylalanal with a chemical shift near 15 ppm [157].

A stark contrast can be discerned in the binding patterns at the active site of thrombin, between hirudin and its mimics, and D-NAPAP. The N-terminal head of r-hirudin forms a parallel β-strand with thrombin (residues 214–219) making a non-substrate like interaction. The first three residues at the N-terminus (Ile^1′^-Val^2′^-Tyr^3′^) of hirudin penetrate the active site and aryl binding site where they interact with the S_2_ and S_3_ specificity sites and form proton bridges to His^57^ and Ser^214^, but avoid the the S_1_ binding site of thrombin. A tightly packed central structure can be observed with intramolecular H-bonds, yet these do not present a signal below 12 ppm in the ^1^H NMR spectrum of hirudin alone. Hirunorms bind to the B chain of thrombin and partly block the active site. Also determined in the X-ray structures of the thrombin complexes is the identical geometry of the catalytic triad, the distance and angle between N and O, which are as in native thrombin. The resonances measured between 15.17 and 15.54 ppm are consistent with donor-acceptor distances <2.70 Å, and an angle of >150° which were calculated from chemical shifts as described above [14]. They compare well with the crystallographic data for the donor acceptor distance in many H-bonds between α-thrombin and r-hirudin, RGD-hirudin and hirunorms. Moreover, from the pH profile for the binding of hirudin to thrombin, we calculated pKa values of 7.1, 8.4 and 9.2, which are consistent with the values from the native enzyme for His^57^, the N amino terminal and the amino group of the Ile residue, respectively. Yet, the pH independence of the 15.33 ppm resonance testifies to the independence of the signal from the ionizing residues (His, N-terminal and Ile). The totality of these results safely excludes the active site as the source of a SHB in the hirudin-inhibited thrombin.

**Figure 11:**
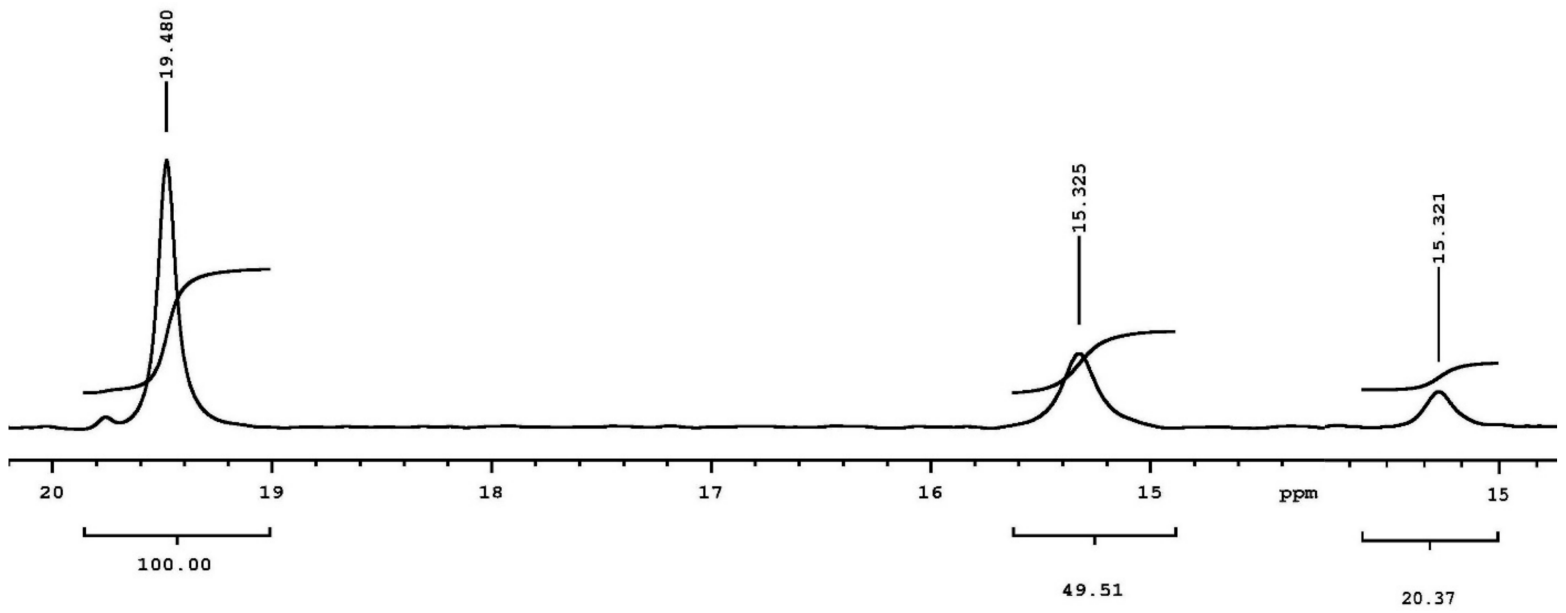
Integration of the resonances obtained for the proton sponge (**left**) and the thrombin hirudin complexes in 6.6% D_2_O (**middle**) and in 58% D_2_O (**right**), both buffered at pH 7.5, 0.30 M HEPES buffer, 0.30 M glycine, 0.10 M NaCl, and 0.005% PEG-8000, 25.0 ± 0.1 °C.

Equilibrium deuterium isotope effects for r-hirudin-inhibited and hirunorm IV and V–inhibited thrombin were measured at pH 7.5 and 6.5 in the established pH-independent range. An example is presented in Figure 11. Fractionation factors between 0.67 and 1.0 were calculated for r-hirudin-inhibited thrombin from the resonances at 15.33 ppm at pH 6.5 in 0.020 M citrate buffer, 0.10 M NaCl and 25.0 and 30.0 ± 0.1 °C. The fractionation factor of 1.0 for the hirunorm V-inhibited thrombin was calculated from the 15.17 ppm resonances under the same conditions. At elevated salt concentrations, above 0.30 M, the fractionation factor is 1.15 for the complexes of thrombin with r-hirudin and RGD-hirudin. The estimated precision in the integration is 20%. These results differ with the fractionation factor of 0.45 ± 0.04 obtained for the active-site inhibited thrombin by PPACK, indicating a shorter proton bridge in an environment of lower dielectric milieu for the latter.

The exchange rates with solvent are smaller than those observed for SHBs in other enzymes [14,80], which reveals a hydrophobic or dry environment of the SHB. It has previously been recognized that contraction of the donor-H-acceptor distance would lead to water exclusion [71].

#### 5.3.5. Sequence Specificity of the ^1^H NMR Resonances in Thrombin-Hirudin Complexes

**Figure 12:**
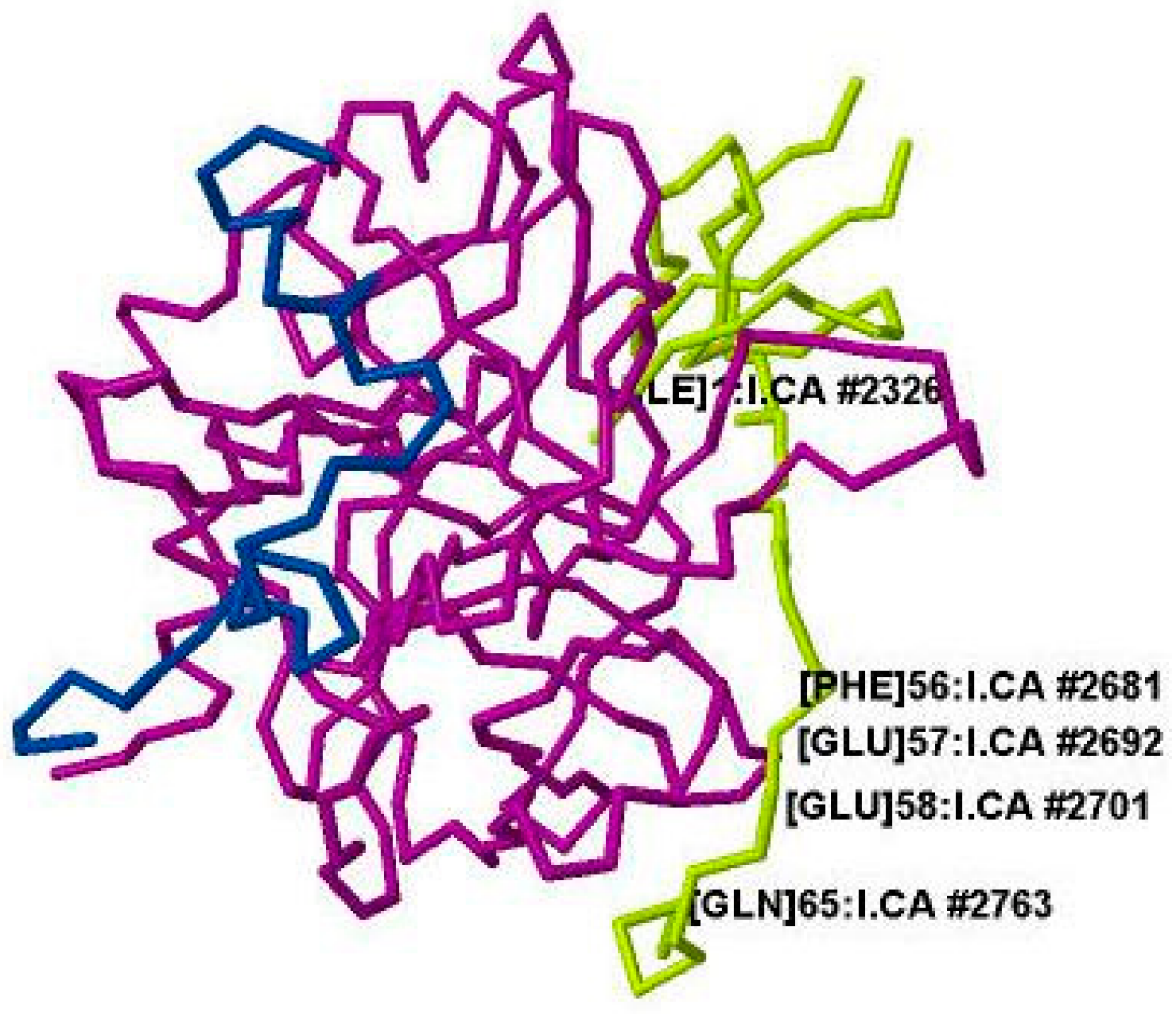
The strand structures of the A chain in blue and B chain in cyan of human-α -thrombin complexed with hirudin in light green. Ile^1′^ and the most likely site of a short hydrogen bond (SHB) occurring in the thrombin-r-hirudin complex, residues 55 to 65 of r-hirudin engaged with Arg^75^ and Arg^77^ of thrombin, are labeled. The image is from the Protein Data Bank PDB file 4HTC [129] modeled with Jmol Version 12.0.41.

In the possession of an arsenal of structural information, the SHB at the interface of thrombin and hirudin should be possible to locate. Three tight-binding regions seemed the most likely candidates for the origin of the SHB giving rise to the 15.33 ppm signal. The binding epitopes on fibrinogen exist in r-hirudin as Ile^1′^, Tyr^3′^ Val^21′^ and the Glu^57′^ Glu^58’^ pair. Our inquiries into the nature of the SHB allows for selective elimination of some of the candidate regions. (1) The emergence of the 18.00 ppm peak after addition of PPACK to the thrombin-hirudin complex, while the 15.33 ppm peak remains, is inconsistent with the existence of an SHB at the active site in the first place. (2) The pH and Na^+^ ion independence of the 15.33 ppm resonance contradicts the presence of an H-bond at the N-terminal amino group of thrombin in the complexes formed between thrombin and the hirudin family of inhibitors. (3) The structures of hirunorms contain a β-(2-naphthyl)alanine at the P_3_ site, which is an unlikely H-bonding partner to H-donors and acceptors at the active site of thrombin and, thus, precludes a SHB forming at this site to give rise to the resonance at 15.17 ppm. (4) Participation of Val^21’^ is abrogated in the hirudin mimics. (5) Consequently, the origin of the SHB is predicated on one or more proton bridges between Glu^57′^ or Glu^58′^ as H acceptors embedded among hydrophobic residues at the C-terminus of hirudin and Arg^75^ and Arg^77^ of thrombin as H donors. In fact, Arg^75^ and Arg^77^ are solvated with water, which are reported to involve H-donor-acceptor distances of 2.6–2.8 Å [128,129]. The most likely site of SHBs is embedded among hydrophobic residues at the C-terminus of hirudin are shown in Figure 12. Such an SHB may result from a compression as the interactions between α-thrombin and hirudin are fine tuned. An SHB between a carboxylate ion and guanidinium ion (Arg) is well supported by the pH independence of the 15.33 ppm peak between pH 5.6 and 8.8.

The X-ray structure of the α-thrombin-(r)sulfohirudin complex was solved at 1.84 Å resolution [55]. The interactions of Tys^63^ in hirudin with α-thrombin are electrostatic with ample opportunities for additional H bonds including SHBs. A prominent interaction is between Tys^63^ in hirudin and Tyr^76^ in thrombin dominated by a H bond between a phenolic OH and an oxygen of Tys^63^. A water molecule also H bonds with the oxygen of Tys^63^, which mediates a water bridge involving another oxygen of the sulfate group, a backbone amide group, and a backbone carbonyl group. Tys^63^ is also in a salt bridge with Lys^81^ in thrombin. This interaction is lacking in α-thrombin modified by the synthetic sulfated-C-terminal peptide, hirugen, containing 10 residues of hirudin [50,158]. Clearly, the sulfate group on hirudin alters the conformation and locks the complex with additional ~3 kcal/mol energy. The totality of interactions, each with small energy, allows for the high potency of native hirudin [37,46]. Presumably, ^1^H NMR studies would reveal if the binding of these regions of sulfated hirudin to thrombin were associated with SHBs and if so what the nature of the resonances is.

## 6. Conclusions

Proton bridges with SHBs eminently contribute to the remarkable catalytic prowess of serine proteases of the blood-clotting cascade and other entities with physiological functions that employ acid-base catalysis. Binding of effectors near the active site elicits conformational adjustments and solvate rearrangements that may or may not exhibit the presence of SHBs. In contrast, these subsite interactions result in the contraction of proton bridges supporting the catalytic apparatus in the chemical steps, which is measurable by the methods delineated in this review. Indeed, the KSIEs under substrate concentrations below enzyme saturation show a component of each an isotopically more and less sensitive step. Conformational adjustments in the binding step are brought to light only by SIEs, proton inventories and thermodynamic studies. When the enzyme is saturated with substrate, the KSIEs become normal between 1 and 4, typically without a contribution of solvent reorganization. The proton inventory technique permits access to intrinsic isotope effects for the masked chemical steps and illuminates the mobilization of (multiple) proton bridges in catalysis by the blood cascade enzymes when specific effectors comply with the stringent requirements for subsite interactions. Fulfillment of the specificity requirements at the P- and P’ sites serve to enforce a compression at the TS to lower the activation energy barrier. When leaving group departure is cumbersome, conformational changes and associated major solvent restructuring occur. The zymogen activation reactions of natural substrates are mediated by solvate reorganization due to interactions between enzyme and substrate at the substrate specificity site or leaving-group binding site, shown in a net increase in the isotopic fractionation factors.

SHBs form at the active site of serine hydrolases (and other enzymes) with transition state analog and mechanism-based inhibitors as PPACK and phosphate or phosphonate inhibitors of thrombin. The SHBs had appeared unique to the attainment of catalytic perfection, but this idea was later questioned [9,82,83,84,159,160,161]. Furthermore, a surprising discovery has been an ^1^H NMR resonance at 18.0 ppm originating not from the catalytic triad in the native rhamnogalacturonan acetylesterase, but from an adjacent SHB between two Asp residues near the oxyanion hole [162]. It became apparent more recently that SHBs are frequent stabilizing elements of tertiary protein structure in compressed regions or in low dielectric fields [163,164]. These are the features that characterize thrombin inhibition with canonical peptide inhibitors, such as D-NAPAP, at the P_1_ and P_2_ binding sites, and even more the non-substrate-like binding inhibitors, for instance, at the allosteric FRS at the thrombin–hirudin interface.

## Figures and Tables

**Chart 1:** The most useful models for fitting proton inventory data.

Information Obtained	Equation
TS_1_	k_n_ = k_H_ (1 − n + n ɸ_1_)
TS_1_, solv.	k_n_ = k_H_ (1 − n + n ɸ_1_) _S_^n^
2TS_1_	k_n_ = k_H_ (1 − n + n ɸ_1_)^2^
2TS_1_, solv.	k_n_ = k_H_ (1 − n + n ɸ_1_)^2^_S_^n^
TS_1_, TS_2_	k_n_ = k_H_ (1 − n + n ɸ_1_)(1 − n + n ɸ_2_)

**Table 1:** Fractionation factors fitting various models for the hydrolysis of N-t-Val-Pro-Arg 7-AMC (amido-4-methylcoumarin) catalyzed by thrombin at pH 8.40, 0.02 M Tris, 0.30 M NaCl, 1% dimethyl sulfoxide (DMSO), 0.1% polyethelene glycole PEG-4000 at 26.0 ± 0.1 °C. Data were fit using explicit weighting by incorporating individual errors [20].

	ɸ_1_	ɸ_2_	ɸ_s_	χ^2^
k_n_/k_H_				
TS_1_	0.32 ± 0.01	----	----	4.997
TS_1_, solv.	0.44 ± 0.05	----	0.70 ± 0.10	2.526
2TS_1_	**0.57 ± 0.01**	**0.57 ± 0.01**	(**1.00**)	2.278
2TS_1_, solv.	0.56 ± 0.04	0.56 ± 0.04	1.04 ±0.15	2.514
TS_1_, TS_2_	0.49 ± 0.15	0.66 ± 0.20	(1.00)	2.515
(k_cat_/K_m_)_n_/(k_cat_/K_m_)_H_				
TS_1_	0.73 ± 0.03	----	----	1.335
TS_1_, solv.	0.42 ± 0.03	----	1.65 ± 0.10	0.274
2TS_1_	0.86 ± 0.02	0.86 ± 0.02	(1.00)	1.514
2TS_1_, solv.	**0.55 ± 0.02**	**0.55 ± 0.02**	**2.4 ±0.2**	0.236
TS_1_, TS_2_	0.47 ± 0.02	1.50 ± 0.06	(1.00)	0.222

**Table 2:** Summary of kinetic solvent isotope effects (KSIEs) and fractionation factors (transition state (TS), ɸ^TS^; solvent, ɸ_S_).

Residues ^1^	^DOD^k_cat_(SD)	ɸ^TS^; ɸ_s_	^DOD^(k_cat_/K_m_)(SD)	ɸ^TS^; ɸ_s_
Thrombin. [20]				
PR	3.27(0.13)	0.38; 0.80	2.09(0.04)	0.38; 1.22
VPR	3.05(0.05)	0.44; 0.70	1.40(0.05)	0.42; 1.65
Or		0.57x ^3^		0.55x2; 2.4
SPR-SFQ	2.24(0.08)	0.44	0.94(0.03)	0.44; 2.4 ^2^
FPR-SFR	2.25(0.24)	0.36x2; 3.1	0.81(0.08)	-; 1.2 ^2^
FVR	2.86(0.06)	0.63x2 ^3^	1.8(0.1)	0.63x2; 1.2
FPiR	2.86(0.07)	0.40x2; 2.0	1.0(0.2)	
*APC* [134]				
IPR	2.35(0.04)	0.60x2 ^3^	1.80(0.01)	
EPR	2.42(0.01)	0.60x2 ^3^	1.90(0.02)	
Plasmin [134]				
EFK	3.48(0.02)	0.60x2 ^3^; 0.7	1.83(0.01)	0.5; 2.1
VFK	2.32(0.02)	0.3; 1.3	1.56(0.01)	0.3; 2.5
VLK	2.76(0.02)	0.2; 1.4	2.52(0.01)	
Factor Xa [22]				
RGR	3.90(0.05)	0.60x2 ^3^; 0.72	1.88(0.03)	0.72x2 ^3^
IPR	2.39(0.53)	0.66x2 ^3^	1.79(0.19)	-

^1^ flanking scissile bond to AMC, lines 1–3; internal quench, lines 4–5; 4-nitoranilide, lines 6–12; ^2^ results confirmed under first-order conditions; ^3^ two identical fractionation factors; standard deviation (SD).

**Table 3:** Summary of kinetic data for the covalent inhibition of human α-thrombin at 25.0 ± 0.1 °C [23].

Inhibitor	pK_a_ 1	pK_a_ 2	k_i_/K_i_, M^−1^ s^−1^ max	k_i_, s^−1^ pH 7.00	K_i_, M pH 7.00	ΔH^‡^ kcal/mol	ΔS^‡^ cal/mol, K
PPACK	7.3 ± 0.2	8.8 ± 0.3	(2.2 ± 0.3) × 10^7^	0.24 ± 0.12	(2.4 ± 1.3) × 10^−8^	10.6 ± 0.7	9 ± 2
Paraoxon	7.8 ± 0.2	9.3 ± 0.3	0.47 ± 0.05		>10^−5^		
NPMP	8.0 ± 0.1	8.6 ± 0.2	6.2 ± 0.1		>10^−5^		

## Abbreviations

7-AMC, 7-amido-4-methylcoumarin; Cha, cyclohexyl-β-alanine; Chg, Cylohexylglycine; DMSO, dimethyl sulfoxide; DSS, 4,4-dimethyl-4-silapentane-1-sulfonic acid sodium salt; EFK-pNA, Pyr-Glu-Phe-Lys-pNA; EPR-pNA, Pyr-Glu-Pro-Arg-pNA; F, blood clotting factor; FPR-SFR, (AB)Val-Phe-Pro-Arg-Ser-Phe-Arg-Leu-Lys(DNP)-Asp-OH; FPiR-pNA, H-D-Phe-L-Pip-Arg-pNA; FRS, fibrinogen recognition site; FVR-pNA, Bz-Phe-Val-Arg-pNA; HPLC, high pressure liquid chromatography; IPR-pNA, H-D-Ile-L-Pro-L-Arg-pNA; KSIE, kinetic solvent isotope effect; LUV, phospholipid vesicles; NPMP, 4-nitrophenyl-2-propyl methylphosphonate; MD, molecular dynamics; MUGB, 4-methylumbelliferyl 4-guanidinobenzoate hydrochloride; NAPAP, Nα-2-naphthylsulfonyl-glycyl-DL-4-amidinophenylalanyl-piperidide acetate; Nα(Me)Arg-peptide,DCha-Pro-Nα(Me)Arg-Thr-(Gly)_5_-^10^Asp-Tyr-Glu-Pro-Ile-Pro-(Glu)_2_-Ala-Cha-^20^DGlu; Paraoxon, 4-nitrophenyl diethyl phosphate; PC, protein C; Pip, piperidyl; PPACK, Phe-Pro-Arg-Chloromethylketone; PR-AMC, Z-Pro-Arg- 7-AMC; QM, quantum mechanical; r-hirudin, recombinant hirudin type 1 or 2; r-RGD-hirudin, recombinant ^32^SGD^34^ type 2 hirudin; RGR-pNA, N-α-Z-D-Arg-Gly-Arg-pNA; RS, reactant state; SHB, short hydrogen bond; SSHB, short strong hydrogen bond; SPR-SFQ, (AB)Val-Ser-Pro-Arg-Ser-Phe-Gln-Lys(DNP)-Asp-OH; TLC, thin layer chromatography_;_ TM, thrombomodulin; TS, transition state; VFK-pNA, H-D-Val-Phe-Lys-pNA; VLK-pNA, H-D-Val-Leu-Lys-pNA; VPR-AMC, N-t-Boc-Val-Pro-Arg-7-AMC.

## Appendix A. Experimental Methods Employed in the Investigations of Short Proton Bridges in the Blood-Clotting Enzymes

### Appendix A.1. Materials Used

Human α-thrombin, TM, antithrombin, prothrombin, human factors Xa and Va, PC, plasmin, porcine trypsin, r-hirudin type1 and type 2, substrates, inhibitors and other chemicals were commercial products of highest purity available (95–99%). Internally fluorescence-quenched substrates were custom synthesized by BioSynthesis Inc Lewisville, TX. Fibrinogen and fibrinopeptides A (FpA) and B (FpB), were also 98% purity but further purified by HPLC as needed and the possibility of interference of the biochemicals and adjuvants in the assays were excluded in control experiments. We received generous gifts: RGD-hirudin [51] from Prof. Wei Mo Molecular Medicine, Ministry of Education, Fudan University, Shanghai China, hirunorms IV and V [53,54] from the late Prof. Vincenzo Pavone University of Padova, Napoli Italy] and DCha-Pro-Nα(Me)Arg-Thr-(Gly)_5_-^10^Asp-Tyr-Glu-Pro-Ile-Pro-(Glu)_2_-Ala-Cha-^20^DGlu (Nα(Me)Arg-peptide) [52] from Prof. Torsten Steinmetzer, Institute of Pharmaceutical Chemistry, Philipps Universitat, Marburg, Germany. 4-Nitrophenyl-2-propyl methylphosphonate (NPMP) was synthesized in this lab previously [20,23,134].

### Appendix A.2. Solvent Isotope Effect and Proton Inventory Measurements and Techniques

Enzyme activity was monitored before and after each experiment using active-site titrants or efficient chromogenic or fluorogenic substrate reactions with previously determined k_cat_ values [20,21,22,23]. Spectrophotometric or fluorimetric assays were used to monitor leaving group departure from substrates, or detection of fluorescence produced by substrate hydrolysis. All kinetic measurements were carried out in identically and carefully prepared buffer solutions in freshly distilled H_2_O and 99% D_2_O to characterize the pH dependence of the kinetic parameters. Depending on the number and the nature of the participating enzyme acid and base residues, at the TS for the rate-limiting step associated with a kinetic parameter, the dependence of the rate constant as a function of pH can be sigmoid, bell-shaped or occasionally more complicated. The pH independent kinetic parameters (k_lim1_ and k_lim2_) and K_a_ of the catalyzing acid or base can be calculated from a fit of the observed rate parameter (k_cat_/K_m_ or k_cat_) to [H^+^] in the general form:

k = K_a_k_lim1_/(K_a_ + [H^+^]) + k_lim2_ (A1)

The pD (pD = electrode reading + 0.4) dependence of the reaction in D_2_O was determined in an identical manner, and thus, the solvent isotope effects on the pH-independent parameters and pKs were calculated.

Complete equilibration in D_2_O at exchangeable catalytic sites were observed with serine proteases under typical experimental conditions and checked in control experiments. Temperatures were maintained within 0.01 °C by appropriate incubation time of all reaction mixtures. The KSIEs were measured first, then mixtures of the buffered isotopic waters were made and the kinetic parameters were determined at a single pH in the pH-independent range of the reactions where k_2_ (less likely k_3_) is rate determining. The highest precision was obtained if the initial rate ratios of the velocity in water to that of a particular atom fraction of D (n), V_i,o_/V_i,n,_ were measured as a function of fractional saturation of enzyme with substrate (f_A_ = [S]/(K_m_ + [S]) = 1/(C_Vf_ + 1)). The K_m_ value measured in water, C_Vf,_ is the commitment factor toward product formation [4,5,6,59] and the partial solvent isotope effects for the Michaelis–Menten parameters were calculated from; V_i,0_/V_i,n_ = f_A_ (^DOD^k_cat_/^n^k_cat_) + (1 − f_A_)(^DOD^k_cat_/K_m_)/(^n^k_cat_/K_m_) [65]. FXa, ACP and plasmin-catalyzed reactions were performed at substrate concentrations saturating the enzyme and followed in full progress curves using the integrated Michaelis Menten Equation (A2) [135,136]:

(A − Ao)/εk_cat_[E} + K_m_/k_cat_(ln(Ainf − Ao)/(Ainf − A)) = t (A2)

where A, Ao and Ainf are the absorbance values at time t, 0 and infinity, respectively; ε is the molar absorptivity, [E] is the enzyme concentration, and k_cat_ and K_m_ are the Michaelis-Menten parameters. Equation (A2) was fitted to two sets of 1000 data points from full progress curves. The calculated k_cat_ values for the reaction in each of nine isotopic buffers were used for constructing the proton inventory curve. The Michaeli–Menten parameters calculated were in good agreement with initial estimates from the first 10–50 data points and with published values under similar conditions. Specific models for protonic participation at the TS_i_ (or solvation) of the rate-determining step *vide infra* were used. Results of the goodness of fit were carefully evaluated and the statistically most feasible model(s) identified.

### Appendix A.3. Experiments for Fibrinogen Activation

The reactions were studied under optimal conditions outlined above in the presence of 5–500 mM NaCl [21]. The reaction was run at fibrinogen concentrations 0.5–30 nM around K_m_ with <0.2 nM thrombin. The rates were adjusted at the working temperature, 37 °C, for convenient sampling at >30 s intervals. The reaction was terminated by the addition of HClO_4_ (~0.3 M). Analysis of the supernatant of the centrifuged solution of the re-dispersed protein were by HPLC using C18 10 µ resin in analytical columns. Elution of the peptides were with 0.02 M pH ~6 buffers using acetonitrile for a linear gradient. In other experiments the turbidity emanating from fibrin formation was monitored by light scatter at 350 nm.

### Appendix A.4. Experiments for Protein C (PC) Activation

The experiments were carried out in properly buffered isotopic waters in the presence of 5–200 mM NaCl and the required amount of choline chloride to keep the salt concentration at 200 mM and in the presence of ~10 nM TM [21]. Aliquots were drawn as a function of time for quenching with r-hirudin 99% of thrombin activity and assaying for emerging APC [118] with a chromogenic substrate of APC under conditions identical to that in water for all samples. The concentrations of APC were measured by initial rates of substrate hydrolysis, spectroscopic monitoring p-NA release at 405 nm and in reference to a previously constructed standard curve.

### Appendix A.5. Experiments for the Inactivation of Thrombin by Active-Site Inhibitors

The inhibition of thrombin with Phe-Pro-Arg-chloromethylketone (PPACK) was monitored by discontinuous sampling for remaining activity as a function of time [23]. Pseudo-first-order rate constants were obtained by fitting the exponential function to the remaining thrombin activity versus time of inhibition for 10 successive incubation times. Inactivation of thrombin by known concentrations of 4-nitrophenyl diethyl phosphate (paraoxon) and NPMP required longer incubation times at constant temperature. Aliquots were drawn from the inhibition mixture and sampled for remaining activity by diluting into the chromogenic assay mixture. Pseudo-first–order rate constants for inhibition were calculated for four half lives from which the second order rate constants were derived. The irreversible inhibition of thrombin by the covalent inhibitors ([I]) were modeled using the following Equation (A3) [23]:

 K_i_        k_i_ E + I  EI EI* (A3)

In this model K_i_ is an equilibrium constant, k_off_/k_on_, where k_on_ is the rate constant for the association of the enzyme and the inhibitor to form the enzyme inhibitor complex, EI, while k_off_ is the rate constant for the dissociation of the enzyme and inhibitor from the EI complex. After the EI complex is formed, k_i_ is the first-order rate constant for bond formation between the enzyme and inhibitor to result in EI*, the covalently modified thrombin. The rate constant observed in the experiments has a hyperbolic dependence on the concentration of I (Equation (A4)) as in the Michaelis–Menten equation [58];

k_obs_ = (k_i_ [I])/(K_i_ + [I]), if [I] << K_i_ the equation simplifies to k_obs_/[I] = k_i_/K_i_ (A4)

This is the formula used to obtain k_i_/K_i_, the second order rate constant, for the encounter of the enzyme and inhibitor to form the EI* complex, as the concentration of PPACK used was much less than the actual K_i_. The pH dependence of the pseudo-first-order rate constant at 2.2 × 10^−9^ M PPACK and 25.0 ± 0.1 °C was plotted and evaluated according to a two-pKa model [3], shown in Equation (A5), that provided the highest probability.

k_obs_ = [I]/(1 + 10^(pK1–pH)^ + 10^(pH–pKI)^) (A5)

All fitting was at the 95% confidence level using simple robust or explicit error propagation (from the inverse of the errors associated with the dependent variable).

### Appendix A.6. Prothrombin Activation Catalyzed by Human FXa

Well-tested methods [22,137,138] were used for the preparation of phospholipid vesicles (LUV) using phosphatidylcholine (Ph). A stock solution was stored frozen and diluted before use to 56 μM LUV, followed by extrusion before use.

For pseudo-first-order kinetic studies, at 5.0 μM prothrombin solution was prepared in pH 7.50 buffer containing 5 × 10^−3^ M CaCl_2_. For prothrombin activation in the presence of LUV, 355 μL of 56 μM LUV was added to a 5 μL of solution of 25 nM FXa and 100 nM FVa to attain a final concentration of 0.33 nM of FXa. The mixture was incubated at 25.0 ± 0.1 °C for 15 min and 15 μL of 5.0 μM prothrombin, to reach 0.20 μM in the prothrombin complex (360 μL), initiated the reaction. Reactions were also carried out with higher FXa concentrations and at different prothrombin concentrations, but in the absence of FVa and LUV.

For initial rate measurements, 41.7 nM FXa (FXa:FVa = 1:4), in pH 7.50 buffer, 0.15 M NaCl and 5 × 10^−3^ M CaCl_2_ was prepared with 56 μM LUV, aliquoted at 10 μL volume, and kept at −20 °C. Prior to the reaction, 10 μL FXa solution was mixed with 110 μL of 56 μM LUV and incubated at 25.0 ± 0.1 °C for 15 min. The reaction was initiated by adding 5 μL of 100 μM prothrombin solution to obtain 4.0 μM prothrombin and 3.3 nM FXa in the prothrombinase complex solution. Reactions were also carried out at different FVa concentrations and at different prothrombin concentrations at 50 μM LUV. In all cases, an aliquot of 20 μL was drawn at appropriate time intervals to test the amidolytic activity of thrombin produced. The sample was added to 715 μL of pH 7.50 buffer and 0.15 M NaCl and preincubated in a cell at 25.0 ± 0.1 °C for 10 min. Fifteen microliters of 3.2 × 10^−3^ M H-D-Phe-L-Pip-Arg-pNA (FPiR-pNA) in pH 7.5 buffer containing 900 nM Soybean trypsin inhibitor and 0.15 M NaCl was added to the solution to start the hydrolysis and pNA release was monitored for one min at 405 nm. The thrombin concentration was calculated from the average of slopes of three to five parallel runs and the k_cat_ value for FPiR-Pna [20]. Repeats from several fresh LUV preparations gave values within 10%. Prothrombin conversion to thrombin occurred near 90%.

### Appendix A.7. Low-Field 1H NMR Protocol

We prepared solutions of 0.2–0.5 mM human α-thrombin with three-fold excess of PPACK or 10-fold excess of 4-nitrophenyl diethylphosphate (paraoxon) or NPMP in pH 6.7, 0.02 M citrate buffer, 0.01 M phosphate buffer, 0.20 M NaCl, 0.1% PEG-8000 [23] and a > five-fold excess was used with Nα-2-naphthylsulfonyl-glycyl-DL-4-amidinophenylalanyl-piperidide acetate salt; (D-NAPAP) at pH 6.5 in 0.05 M sodium citrate buffer, or in 0.01 M phosphate buffer (pH = 6.5), 0.20 M NaCl, 0.1% PEG8000 [26]. R-hirudin or its analogs and the Nα(Me)Arg-peptides were used in slight excess over thrombin concentrations. Identical concentrations and conditions were used for samples of, r-hirudin and hirunorm V for control [26]. The effect of Na^+^ ion, buffer composition and temperature were probed with thrombin-r-hirudin and RGD-hirudin complexes in pH 6.5, 0.02–0.04 M citrate or 0.04–0.10 M phosphate buffer which contained NaCl between 0.09 and 0.31 M concentrations. The D_2_O content was ~7% in general and ~45–55% for the isotope effect studies. A 99% loss of thrombin activity was achieved with PPACK and 90% loss of activity was obtained with paraoxon and NPMP, 97% and 98% activity loss were observed with the Nα(Me)Arg-peptide and D-NAPAP inhibition, respectively, and >99% loss of activity was achieved with r-hirudin, and its mimics. Trypsin samples were made in 1.8 mM concentration with and without ~3-fold excess of D-NAPAP in pH 7.00, 0.10 M phosphate buffer, 0.1% PEG-4000 and 7% D_2_O. Inhibition was >70%. We probed the ^1^H NMR samples for protein aggregation by polyacrylamide gel electrophoresis (PAGE). Only one band corresponding to the 36,500 Da of thrombin was obtained.

A 5 mm triple resonance probe was used, water excitation was avoided by using a 1331 pulse sequence and a 90° pulse width of 30 μs for a 512 ms acquisition time including a 2.5 s relaxation delay were applied. We allowed the phosphate and phosphonate ester-inhibited thrombin samples to “age” for 50 h and then reexamined them. To determine the D/H fractionation factors from deshielded resonances at maximal sensitivity the samples were divided into two, one in buffered H_2_O and the other in identically buffered D_2_O. The proton sponge 1,8-bis(dimethylamino) naphthalene was dissolved in CD_3_CN then titrated with H_2_SO_4_. It was placed in a capillary to serve as an external chemical shift reference for quantitation of deshielded resonances. One-dimensional ^1^H NMR spectra were acquired at 30 °C with 3000–16,000 transients for free thrombin, free r-hirudin, free hirunorm V, covalently modified thrombin with PPACK and for complexes of thrombin with D-NAPAP, r-hirudin with or without PPACK, and for complexes of thrombin with hirudin mimics. The chemical shifts are reported relative to low field from external DSS. Temperature studies of the resonances were in the 5–35 °C range. From the integrated signals in 7% and 55% D_2_O buffers the fractionation factors (ɸ) were calculated as follows: I = [Imax(X)] ɸ/(1 − X) + X], where X = mole fraction of H_2_O; I = observed intensity and Imax is maximal intensity at X = 1.0.
